# Factors associated with delays in assessment and treatment of obsessive-compulsive disorder: A scoping review

**DOI:** 10.1016/j.jocrd.2025.100982

**Published:** 2025-10-27

**Authors:** Kaeo Wongbusarakum, Erica Schug, Tamerlane C. Visher, Kaitlyn Sulivan-Pascual, Megan Mirkis, Precia Rhee, Adam C. Frank

**Affiliations:** a University of Southern California, 3551 Trousdale Parkway, Los Angeles, CA, 90089, USA; b Drexel University College of Medicine, 60 N 36th St, Philadelphia, PA, 19104, USA; c Keck School of Medicine of USC, Department of Psychiatry and Behavioral Sciences, 2250 Alcazar St # 2200, Los Angeles, CA, 90033, USA

## 1. Introduction

### 1.1. Background

Obsessive-Compulsive Disorder (OCD) is a neuropsychiatric condition with a lifetime prevalence of around 2 % (Pampaloni et al., 2022; Ruscio et al., 2010). OCD impacts the quality of life of those afflicted: individuals can spend hours each day on obsessions and compulsions, contributing to lost days of work and strained professional and personal relationships (Moritz, 2008). First-line treatment for OCD includes pharmacotherapy with selective-serotonin reuptake inhibitors (SSRIs), psychotherapy with exposure and response prevention (ERP), or a combination of these approaches; for those that do not benefit from first-line treatment options, neuroleptics and neuromodulation are also offered (Hirschtritt et al., 2017).

Despite OCD’s prevalence, impact on quality of life, and the availability of evidence-based treatments, there are significant delays between the onset of OCD symptoms, diagnosis, and adequate treatment, often exceeding 7 years, and historically estimated to be over 17 years (Albert et al., 2019b; Hezel et al., 2022; Hollander et al., 1997). Studies have consistently shown that longer duration from symptom onset to treatment portends worse treatment outcomes (Dell’Osso et al., 2010; Catapano et al., 2006; Wootton, 2016). Additionally, research has also suggested that longer duration of untreated OCD is associated with greater symptom severity (Perris et al., 2023; Wootton, 2016), the presence of more comorbid conditions (Pellegrini et al., 2025) and even certain symptoms clusters (Zheng et al., 2021). While individual studies have attempted to explore these factors, there is limited summative research focusing on factors related to delays in diagnosis and treatment of OCD.

As of July 2023, there are two reviews in English on factors associated with delayed treatment or treatment-seeking behavior in OCD: a review of 12 studies conducted in 2014 (García-Soriano et al., 2014) and a US-centric review of 27 studies conducted in 2021 (Senter et al., 2021). In the first review of 12 studies, treatment-seeking was tied to greater awareness of the disorder, greater severity of the disorder, and the presence of comorbid mental disorders (García-Soriano et al., 2014). In the second review conducted in 2021 (Senter et al., 2021), inadequate treatment was tied to misdiagnosis of the disorder, inadequate use of SSRIs, and inadequate use of exposure and response prevention therapy (Senter et al., 2021). As of July 2023, no reviews have focused on factors related to delays in diagnosis and treatment of OCD with an international scope.

### 1.2. Objective

To improve recognition of OCD and subsequent access to high-quality mental healthcare, it is important to increase our understanding of the factors related to delays in diagnosis and treatment of OCD. In this scoping review, we map the extant English-language literature from around the world on factors associated with delays in diagnosis and treatment of OCD.

## 2. Methods

### 2.1. Study design

In line with the study objectives to understand the nature and scope of the existing literature on factors related to delays in diagnosis and treatment of OCD, a scoping review was chosen as the most appropriate synthesis approach. The purpose of a scoping review is to map the breadth and depth of research activity in a specific area, particularly when topics are emerging or insufficiently explored. This method allows for a systematic assessment of available evidence and identification of key concepts and gaps in the literature (Munn et al., 2018).

The protocol for this review was preregistered at the Open Science Foundation on July 17, 2023 (Visher et al., 2023). PRISMA-ScR (Preferred Reporting Items for Systematic Reviews and Meta-Analyses extension for Scoping Reviews) guidelines were followed for the execution of this scoping review. The search was conducted between July 20, 2023 and July 27, 2023 across four databases: PubMed, EMBASE, APA PsycInfo, and Web of Science. Keywords were drawn from the literature and refined through discussions with the research team. The final PubMed search strategy was: (“OCD” OR “obsessive” OR “obsessive-compulsive”) AND (“time-to-treatment” OR “delayed” OR “delayed treatment” OR “delayed diagnosis” OR “stigma” OR “accommodation” OR “family accommodation” OR “awareness” OR “untreated” OR “duration of untreated illness” OR “early intervention” OR “early age of onset” OR “help-seeking”). Appropriate search strategy modifications were made for analogous searches in EMBASE, APA PsycInfo, and Web of Science. Relevant studies were entered into Covidence (Veritas Health Innovation) for screening and data extraction, and duplicates were removed using automated processes.

### 2.2. Inclusion and exclusion criteria

To ensure comprehensive inclusion of relevant studies, we used the Population, Concept, and Context framework as outlined in the Joanna Briggs Institute Reviewers’ Manual for scoping reviews (Peters et al., 2015). Inclusion criteria for the study population are as follows: individuals with OCD as the primary diagnosis and participants of any age, sex, or geographical location. We also included studies that encompassed individuals with other psychiatric conditions, as long as a subset of the participants had OCD as the primary diagnosis. Studies that did not include individuals with OCD as the primary diagnosis were excluded. Inclusion criteria for the study concept are as follows: studies that specifically address factors contributing to delays in OCD diagnosis or treatment. These factors include but are not limited to stigma, family accommodation, awareness, cultural factors, and other barriers impacting timely diagnosis and access to evidence-based treatment. Inclusion criteria for the study context are as follows: peer-reviewed studies conducted in diverse care environments, including outpatient clinics, inpatient hospitals, community-based care, and other relevant settings. Only studies published in English were included to ensure consistent and thorough data extraction and interpretation.

To align with the standards of a scoping review, we excluded review articles, meta-analyses, conference abstracts, and thesis defenses, as these publication types often lack the primary data needed to map the evidence landscape effectively and may not be peer-reviewed. There were no restrictions on publication date, allowing for an inclusive exploration of the available literature on this topic.

All studies were screened independently by 2 reviewers using Covidence. Conflicts in screening were resolved through discussion to ensure consensus. Following screening, full texts of relevant records were obtained. Two authors independently extracted data and reached consensus on the final extracted items through joint discussions with ACF.

### 2.3. Development of themes and factors

The themes and factors were developed through an iterative process. Following each consensus meeting, team members identified factors that were associated with delays in care for each study, which were recorded in a table. After every study had been reviewed, team members revisited the table and discussed general findings. Related factors were then grouped together to comprise a theme, which resulted in 6 overarching themes, with each composed of specific related factors that led to delays in OCD care. Some studies uncovered factors that improved timely access to diagnosis and care and these factors were also included in the table and reviewed iteratively. This approach was effective in capturing recurring factors in studies and contextualizing them within broader themes. All factors and themes emerged inductively from the data and discussion among study team members and were not predetermined at study outset.

## 3. Results

### 3.1. Section 1: overview

**Fig. 1.**
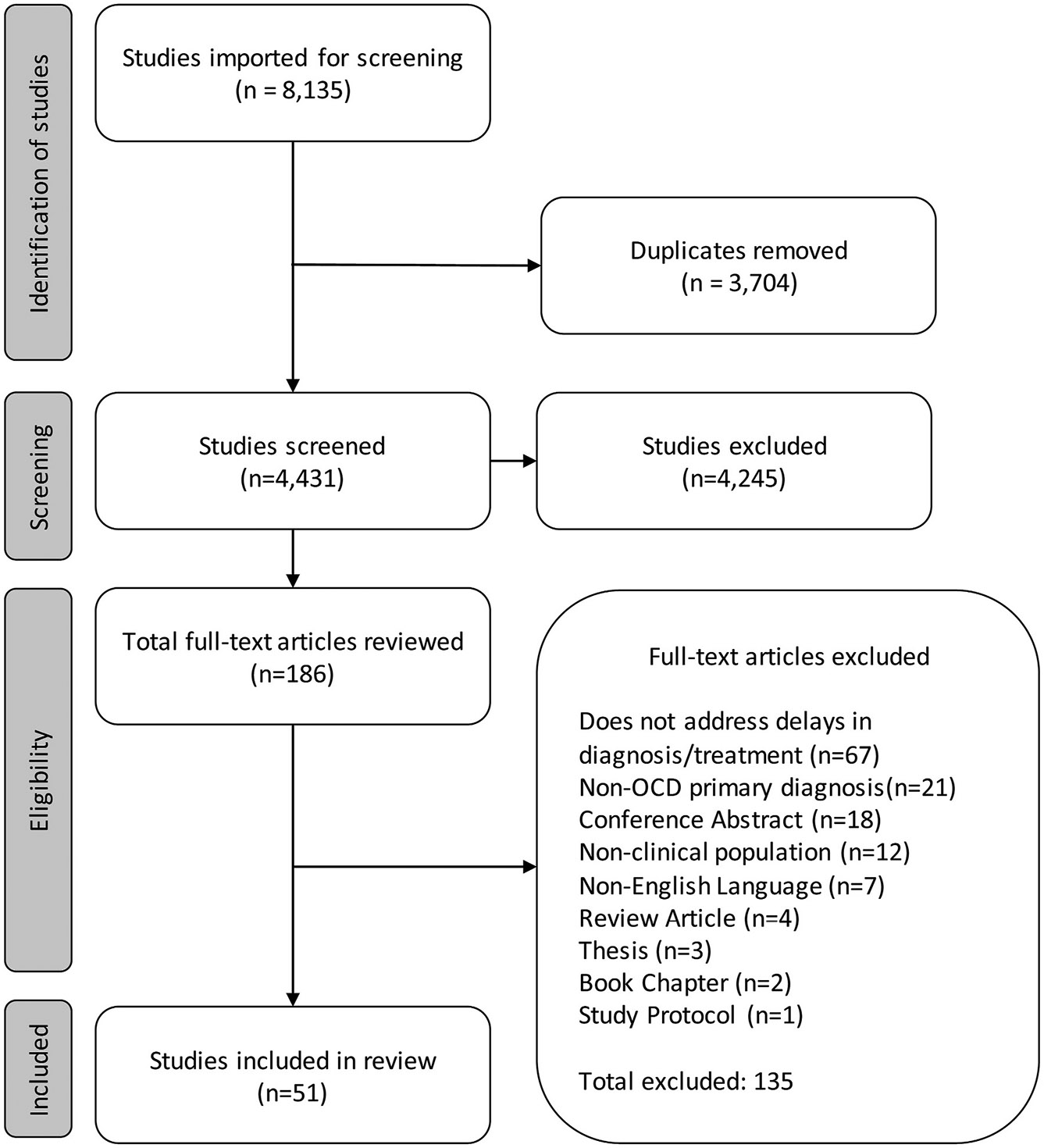
PRISMA (Preferred Reporting Items for Systematic Reviews and Meta-Analyses) Diagram. OCD: obsessive-compulsive disorder.

This scoping review was conducted using the Covidence review software, which allows for the automatic removal of duplicate records, blinded screening, extraction by individual reviewers, and systematic tracking of studies. Fig. 1 displays the PRISMA diagram. A total of 8135 studies were identified. Following the removal of duplicates (3704/8135, 45.53 % of total studies), 4431 (54.47 %) studies remained for title and abstract screening. After screening, 4245/4431 (95.80 %) studies were excluded for not being relevant to the review’s research question. The remaining 186 (2.29 % of total studies) studies underwent full-text review, during which an additional 135/186 (72.58 %) studies were excluded for not meeting inclusion/exclusion criteria, leaving 51 studies that underwent data extraction and are included herein.

Of the 51 studies, there were 10 qualitative studies, 39 quantitative studies, and 2 mixed methods studies. While a majority (43) of studies included participants who had a diagnosis of OCD (Gentle et al., 2014), (Mahintorabi et al., 2017), (Vigne et al., 2019), (Costa et al., 2022), (Zheng et al., 2021), (Okasha et al., 2021), (Stengler et al., 2013), (Ziegler et al., 2021), (Wahl et al., 2010), (Altamura et al., 2010), (Torres et al., 2007), (Chakraborty et al., 2013), (Viswanath et al., 2011), (Grover et al., 2014), (Benatti et al., 2016), (Dell’Osso et al., 2015),^174^, (Perris et al., 2021), (Albert, Barbaro, et al., 2019), (Matsumoto et al., 2021), (Al-Solaim & Loewenthal, 2011), (Hathorn et al., 2021), (Del Valle et al., 2018), (Del Valle et al., 2017), (Belloch et al., 2009), (Besiroglu et al., 2004), (Karadaĝ et al., 2006), (Poyraz et al., 2015), (Fernández de la Cruz et al., 2015), (Vuong et al., 2016), (Pedley et al., 2019a), (Robinson et al., 2017), (Keyes et al., 2018), (Pedley et al., 2019b), (Koran et al., 2000), (Hezel et al., 2022), (Williams et al., 2012), (Grant et al., 2007), (Swisher et al., 2023), (Marques et al., 2010), (Levy et al., 2013), (Waumans et al., 2022), there were also 4 studies that used a geographic population as a sample and did not limit participants to those with an existing OCD diagnosis (Caraveo-Anduaga and Bermúdez, 2004; Chong et al., 2012; Fullana et al., 2009; Subramaniam et al., 2020) . Three studies contained participant samples that did not have OCD but instead were family members of those with OCD (Chessell et al., 2023; Kolvenbach et al., 2018; Stengler-Wenzke et al., 2004). There was 1 case study (Ferreira et al., 2021) that included a single patient with OCD. Thirteen studies had sample sizes ranging from 1 to 50 patients (Waumans et al., 2022),(Al-Solaim & Loewenthal, 2011), (Besiroglu et al., 2004), (Chessell et al., 2023). (Mahintorabi et al., 2017), (Ferreira et al., 2021), (Robinson et al., 2017), (Belloch et al., 2009), (Kolvenbach et al., 2018), (Stengler et al., 2013), (Keyes et al., 2018), (Pedley et al., 2019a), (Hathorn et al., 2021); 21 had sample sizes ranging from 51 to 200 participants (Stengler et al., 2013; Chakraborty et al., 2013; Hezel et al., 2022; Gentle et al., 2014; Vuong et al., 2016; Del Valle et al., 2018; Williams et al., 2012; Karadaĝ et al., 2006; Viswanath et al., 2011; Benatti et al., 2016; Dell’Osso et al., 2015; Ziegler et al., 2021; Swisher et al., 2023; Del Valle et al., 2017; Dell’Osso et al., 2015; Okasha et al., 2021; Poyraz et al., 2015; Vigne et al., 2019; Marques et al., 2010; Perris et al., 2021; Matsumoto et al., 2021; Grover et al., 2014); and 16 had sample sizes greater than 200 participants (Albert, Barbaro, et al., 2019; Altamura et al., 2010; Caraveo-Anduaga and Bermúdez, 2004; Chong et al., 2012; Costa et al., 2022; Cullen et al., 2008; Fernández de la Cruz et al., 2015; Fullana et al., 2009; Grant et al., 2007; Koran et al., 2000; Levy et al., 2013; Pedley et al., 2019b; Subramaniam et al., 2020; Torres et al., 2007; Wahl et al., 2010; Zheng et al., 2021). The largest sample size was 6126 participants (Chong et al., 2012) and was composed of Singapore citizens and permanent residents aged 18 years and older. The countries from which the studies were conducted included Australia (Gentle et al., 2014; Mahintorabi et al., 2017), Brazil (Costa et al., 2022; Vigne et al., 2019), China (Zheng et al., 2021), Egypt (Okasha et al., 2021), Germany (Stengler et al., 2013; Stengler-Wenzke et al., 2004; Wahl et al., 2010; Ziegler et al., 2021), India (Chakraborty et al., 2013; Grover et al., 2014; Viswanath et al., 2011), Italy (Benatti et al., 2016; Dell’Osso et al., 2015; Dell’Osso et al., 2017; Altamura et al., 2010; Perris et al., 2021; Albert, Barbaro, et al., 2019), Japan (Matsumoto et al., 2021), Mexico (Caraveo-Anduaga and Bermúdez, 2004), New Zealand (Fullana et al., 2009), Portugal (Ferreira et al., 2021), Saudi Arabia (Al-Solaim & Loewenthal, 2011), Singapore (Chong et al., 2012; Subramaniam et al., 2020), South Africa (Hathorn et al., 2021), Spain (Belloch et al., 2009; Del Valle et al., 2017, 2018), The Netherlands (Waumans et al., 2022), Turkey (Besiroglu et al., 2004; Karadaĝ et al., 2006; Poyraz et al., 2015), the United Kingdom (Chessell et al., 2023; Fernández de la Cruz et al., 2015; Keyes et al., 2018; Kolvenbach et al., 2018; Pedley et al., 2019a, 2019b; Robinson et al., 2017; Torres et al., 2007; Vuong et al., 2016), and the United States (Cullen et al., 2008; Grant et al., 2007; Hezel et al., 2022; Koran et al., 2000; Levy et al., 2013; Marques et al., 2010; Swisher et al., 2023; Williams et al., 2012). Nearly all of the studies were restricted to an adult population, but 12 of studies included participants under the age of 18 (Costa et al., 2022), (Stengler-Wenzke et al., 2004), (Torres et al., 2007), (Matsumoto et al., 2021), (Karadaĝ et al., 2006), (Fernández de la Cruz et al., 2015), (Pedley et al., 2019a), (Pedley et al., 2019b), (Kolvenbach et al., 2018), (Keyes et al., 2018), (Koran et al., 2000), (Cullen et al., 2008), and 8 did not specify age range nor age restrictions for participants (Al-Solaim & Loewenthal, 2011; Altamura et al., 2010; Benatti et al., 2016; Fullana et al., 2009; Grover et al., 2014; Viswanath et al., 2011; Wahl et al., 2010; Zheng et al., 2021).

**Fig. 2.**
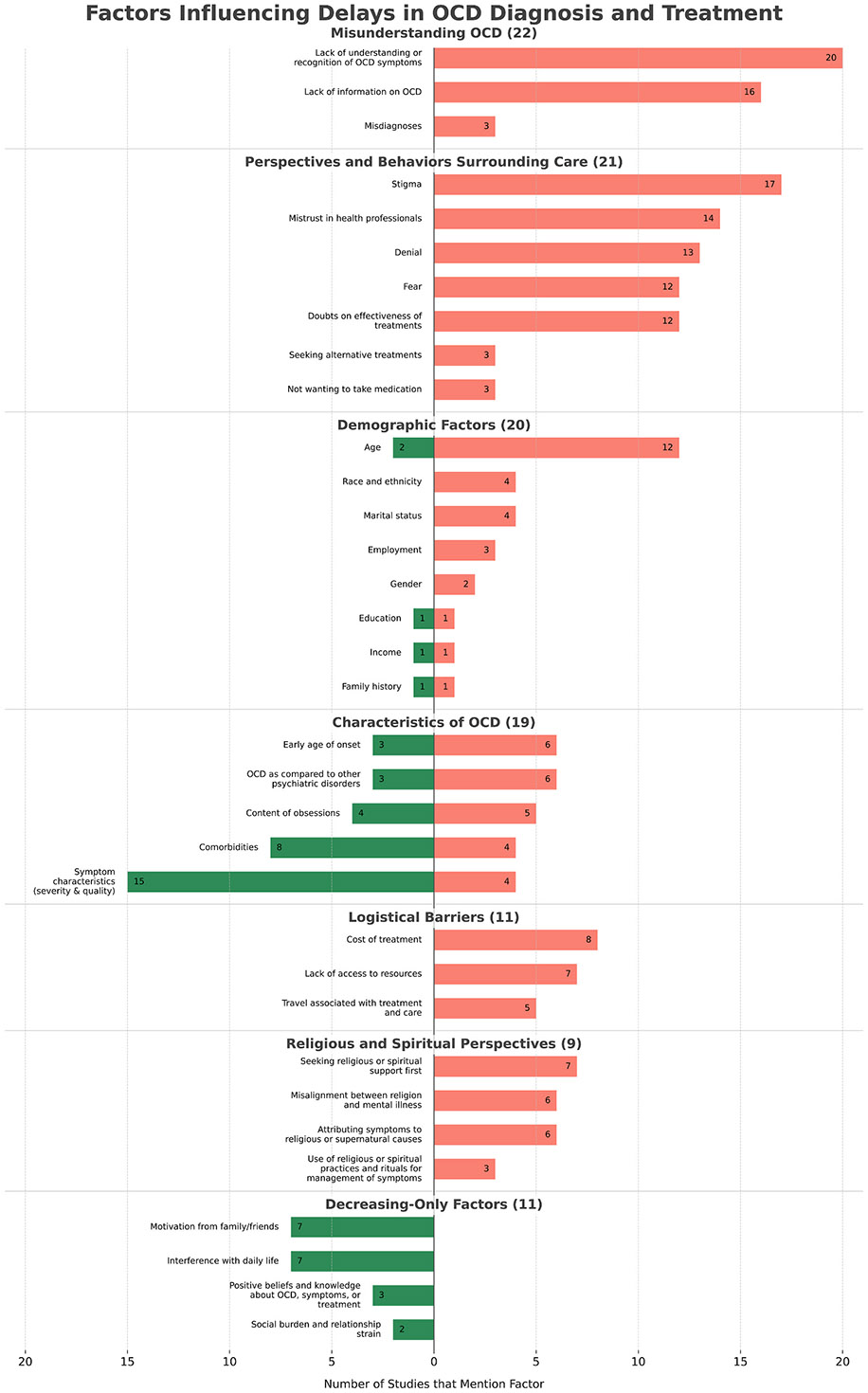
Factors Influencing Delays in OCD Diagnosis and Treatment. Bar graph showing the number of studies identifying each factor associated with increased or decreased delays. Factors are grouped within their respective themes—except for those exclusively associated with decreased delays—and arranged in descending order according to the number of studies reporting them. Numbers in parentheses in the section headers indicate the total number of studies that identified each theme; as studies can include multiple factors, factor numbers are not strictly additive to studies indicated in parentheses.

Self-identified reasons for delays in OCD care and demographic and clinical factors associated with delays in OCD care are summarized and discussed in the subsequent sections, in Table 1, and in Fig. 2. All factors associated with delays in OCD care were organized into 6 distinct themes: misunderstanding OCD, perspectives and behaviors surrounding care, demographic factors, characteristics of OCD, logistical barriers, and religious and spiritual factors. Some factors were found to be associated with both increases and decreases in delays in care and these are indicated in Fig. 2 and discussed in Section 3. Several factors were exclusively associated with decreased delays in diagnosis and treatment, and were included in their own theme and discussed in Section 3. Finally, some factors were found not to have an association with improving or hindering access to care and these are briefly discussed in Section 4.

### 3.2. Section 2: factors associated with delays in diagnosis and treatment

#### 3.2.1. Section 2.1 theme 2 - misunderstanding OCD

Misunderstanding OCD emerged in 22 studies and consisted of 3 factors: lack of understanding or recognition of OCD symptoms, lack of information on OCD, and misdiagnoses (Al-Solaim & Loewenthal, 2011; Belloch et al., 2009; Chakraborty et al., 2013; Chessell et al., 2023; Del Valle et al., 2017; Ferreira et al., 2021; Gentle et al., 2014; Grover et al., 2014; Keyes et al., 2018; Kolvenbach et al., 2018; Mahintorabi et al., 2017; Marques et al., 2010; Matsumoto et al., 2021; Okasha et al., 2021; Pedley et al., 2019a; Poyraz et al., 2015; Robinson et al., 2017; Stengler-Wenzke et al., 2004; Vuong et al., 2016; Wahl et al., 2010; Waumans et al., 2022; Williams et al., 2012).

Lack of understanding or recognition of OCD symptoms as those of a disorder was identified in 20 studies (Al-Solaim & Loewenthal, 2011; Belloch et al., 2009; Chakraborty et al., 2013; Chessell et al., 2023; Del Valle et al., 2017; Ferreira et al., 2021; Gentle et al., 2014; Grover et al., 2014; Keyes et al., 2018; Kolvenbach et al., 2018; Mahintorabi et al., 2017; Matsumoto et al., 2021; Pedley et al., 2019a; Poyraz et al., 2015; Robinson et al., 2017; Stengler-Wenzke et al., 2004; Vuong et al., 2016; Waumans et al., 2022; Williams et al., 2012). For example, in a study surveying the duration of untreated illness (DUI) of participants in an outpatient clinic located in Japan, 64.5 % of 71 OCD participants delayed hospital visits for treatment because they "did not think OCD symptoms were associated with an illness" (Matsumoto et al., 2021). In the UK, 22 parents of 16 children with OCD described having difficulty distinguishing between challenges associated with natural child development and symptoms of a developing disorder (Chessell et al., 2023). In another study from the UK, some participants perceived their behaviors as consistent with their personality (Pedley et al., 2019a). Finally, in Spain, 50 % of participants reported they were “convinced that the problem was temporary” as a reason for delaying treatment (Belloch et al., 2009).

Lack of information on OCD was identified in 16 studies (Al-Solaim & Loewenthal, 2011; Belloch et al., 2009; Chakraborty et al., 2013; Chessell et al., 2023; Del Valle et al., 2017; Gentle et al., 2014; Grover et al., 2014; Kolvenbach et al., 2018; Mahintorabi et al., 2017; Marques et al., 2010; Pedley et al., 2019a; Poyraz et al., 2015; Robinson et al., 2017; Stengler-Wenzke et al., 2004; Vuong et al., 2016; Williams et al., 2012). Parents of children with OCD from the UK indicated they wanted to help their children but did not know how (Chessell et al., 2023). Similarly, a study from the Netherlands found that limited knowledge was associated with increased delays in treatment (Waumans et al., 2022). Nearly half (47.1 %) of participants in a US-based study reported not knowing they had a disorder or that there were available treatments (Williams et al., 2012). In this same study, 50.00 % of White Americans and 76.06 % of Black Americans were unsure who to see or where to go for treatment (Williams et al., 2012).

Misdiagnoses were identified in 3 studies, reflecting that delays can occur even in standard clinical settings (Stengler-Wenzke et al., 2004; Vuong et al., 2016; Wahl et al., 2010). One study found that OCD was not recognized in more than 70 % of study participants (Wahl et al., 2010). Some participants also cited misdiagnoses from health professionals as contributing to seeking care from alternative sources (Vuong et al., 2016).

#### 3.2.2. Section 2.2 theme 1 - perspectives and behaviors surrounding care

Perspectives and behaviors surrounding care for OCD was a theme in 21 studies (Al-Solaim & Loewenthal, 2011; Belloch et al., 2009; Chessell et al., 2023; Del Valle et al., 2017, 2018; Ferreira et al., 2021; Gentle et al., 2014; Hathorn et al., 2021; Keyes et al., 2018; Kolvenbach et al., 2018; Mahintorabi et al., 2017; Marques et al., 2010; Matsumoto et al., 2021; Okasha et al., 2021; Pedley et al., 2019a; Poyraz et al., 2015; Robinson et al., 2017; Stengler-Wenzke et al., 2004; Vuong et al., 2016; Waumans et al., 2022; Williams et al., 2012). Seven factors were identified: stigma, mistrust in health professionals, denial, fear, doubts on effectiveness of treatments, seeking alternative treatments, misdiagnoses, and not wanting to take medication.

Stigma was identified in 17 studies as a reason for not seeking treatment (Belloch et al., 2009; Del Valle et al., 2017, 2018; Ferreira et al., 2021; Gentle et al., 2014; Hathorn et al., 2021; Keyes et al., 2018; Kolvenbach et al., 2018; Marques et al., 2010; Matsumoto et al., 2021; Okasha et al., 2021; Poyraz et al., 2015; Robinson et al., 2017; Stengler-Wenzke et al., 2004; Vuong et al., 2016; Waumans et al., 2022; Williams et al., 2012). For example, participants endorsed “embarrassment and shame factors” (Hathorn et al., 2021), feeling “worried about others’ opinions” (Marques et al., 2010), or general “stigma and judgment” (Williams et al., 2012). Similarly, family members of participants with OCD also identified stigma with delays in diagnosis (Kolvenbach et al., 2018; Stengler-Wenzke et al., 2004).

Mistrust in health professionals was identified in 14 studies, with participants expressing a lack of trust in healthcare providers and treatment recommendations (Al-Solaim & Loewenthal, 2011; Belloch et al., 2009; Del Valle et al., 2017; Gentle et al., 2014; Keyes et al., 2018; Kolvenbach et al., 2018; Marques et al., 2010; Matsumoto et al., 2021; Pedley et al., 2019a; Poyraz et al., 2015; Robinson et al., 2017; Stengler-Wenzke et al., 2004; Vuong et al., 2016; Williams et al., 2012). Mistrust due to race or ethnicity was also noted, with Black Americans more frequently indicating being “uncomfortable discussing problems with professionals” or fearing they “would be treated unfairly because of race or ethnicity” (Williams et al., 2012). Members of ethnic minority groups were also found to express culturally-based mistrust of the mental health system (Kolvenbach et al., 2018).

Denial was identified in 13 studies with participants expressing refusal to disclose, receive treatment, or admit to a diagnosis (Belloch et al., 2009; Chessell et al., 2023; Del Valle et al., 2017; Gentle et al., 2014; Hathorn et al., 2021; Keyes et al., 2018; Kolvenbach et al., 2018; Mahintorabi et al., 2017; Marques et al., 2010; Matsumoto et al., 2021; Poyraz et al., 2015; Robinson et al., 2017; Williams et al., 2012). Many participants did not seek treatment because they were afraid to have a diagnosis of a mental illness, or they were ashamed of their symptoms (Matsumoto et al., 2021). Some also had the belief that they “could manage or handle symptoms on his/her own” (Gentle et al., 2014; Poyraz et al., 2015).

Fear was identified in 12 studies (Al-Solaim & Loewenthal, 2011; Belloch et al., 2009; Del Valle et al., 2017, 2018; Gentle et al., 2014; Kolvenbach et al., 2018; Marques et al., 2010; Poyraz et al., 2015; Robinson et al., 2017; Stengler-Wenzke et al., 2004; Vuong et al., 2016; Williams et al., 2012). Participants often expressed fear and apprehension toward both treatment and receiving a diagnosis. In one study, 30 % of participants endorsed the fear of being considered a “mentally ill person” (Belloch et al., 2009). Other studies highlighted fear of “being committed to a hospital against my will” (Gentle et al., 2014) or fear of criminalization (Robinson et al., 2017).

Doubts on the effectiveness of treatments were identified in 13 studies (Al-Solaim & Loewenthal, 2011; Belloch et al., 2009; Gentle et al., 2014; Hathorn et al., 2021; Keyes et al., 2018; Kolvenbach et al., 2018; Mahintorabi et al., 2017; Marques et al., 2010; Matsumoto et al., 2021; Pedley et al., 2019a; Poyraz et al., 2015; Vuong et al., 2016; Williams et al., 2012). Participants delayed starting treatment because they believed it would not work for them or would be generally ineffective (Marques et al., 2010; Poyraz et al., 2015; Williams et al., 2012). Additionally, exposure and response prevention (ERP) therapy was perceived as challenging by an adolescent population, and participants expressed resistance and conflict (Keyes et al., 2018). Doubt and hesitation around taking pharmaceutical medication was identified in 3 studies (Matsumoto et al., 2021; Pedley et al., 2019a; Poyraz et al., 2015). Some participants mentioned the “possibility of using medication” as a reason for not seeking treatment (Matsumoto et al., 2021; Poyraz et al., 2015). Others expressed doubt about the effectiveness of medication as a standalone treatment, and did not believe that it would deal with the underlying issues of OCD (Pedley et al., 2019a).

Seeking alternative treatments was identified in 3 studies, with participants seeking help from sources other than healthcare professionals (Matsumoto et al., 2021; Poyraz et al., 2015; Vuong et al., 2016).

#### 3.2.3. Section 2.3 theme 3 - demographic factors

Demographic factors were highlighted in 20 studies, with 8 factors including age, race and ethnicity, marital status, employment, gender, education, income, and family history (Albert, Barbaro, et al., 2019; Caraveo-Anduaga and Bermúdez, 2004; Chong et al., 2012; Costa et al., 2022; Cullen et al., 2008; Gentle et al., 2014; Hezel et al., 2022; Kolvenbach et al., 2018; Koran et al., 2000; Levy et al., 2013; Matsumoto et al., 2021; Perris et al., 2021; Poyraz et al., 2015; Stengler-Wenzke et al., 2004; Subramaniam et al., 2020; Swisher et al., 2023; Viswanath et al., 2011; Williams et al., 2012; Zheng et al., 2021; Ziegler et al., 2021).

Age occurred as a factor in 12 studies (Albert, Barbaro, et al., 2019; Chong et al., 2012; Costa et al., 2022; Cullen et al., 2008; Hezel et al., 2022; Koran et al., 2000; Levy et al., 2013; Matsumoto et al., 2021; Poyraz et al., 2015; Swisher et al., 2023; Zheng et al., 2021; Ziegler et al., 2021). Older age at time of study participation was associated with longer delays in seeking treatment (Costa et al., 2022; Ziegler et al., 2021). Another study found a higher percentage of newly diagnosed children and adolescents (49.4 %) had not received a trial of medication for OCD in the year following diagnosis, compared to newly diagnosed adults (27.5 %) (Koran et al., 2000).

The factor of race and ethnicity was found in 4 studies (Gentle et al., 2014; Kolvenbach et al., 2018; Swisher et al., 2023; Williams et al., 2012). In a US-based study, “does not trust White establishment” was provided as a reason for delaying or avoiding seeking treatment. In the same study, participants endorsed “would be treated unfairly because of race or ethnicity” (White Americans = 7.41 %, Black Americans = 22.54 %, Fisher’s Exact Test, p = 0.006) as a barrier to treatment (Williams et al., 2012). Separately, parents of children with OCD reported reasons such as “stigma and discrimination in family and community” (white group n = 2, ethnic minority group n = 9), and “discrimination within the system” (white group n = 0, ethnic minority group n = 2) as barriers to accessing care (Kolvenbach et al., 2018).

Marital status was identified in 4 studies (Albert, Barbaro, et al., 2019; Cullen et al., 2008; Swisher et al., 2023; Zheng et al., 2021). In a study of 602 adults with OCD, being married was associated with a lack of receiving treatment (treated: 55.9 % married; untreated: 78 % married; *χ*2 = 19.52, df = 2, p < 0.001) (Cullen et al., 2008). Similarly, married participants were found to have significantly longer treatment delays (11.35 years) compared to single participants (5.55 years) (Swisher et al., 2023).

Employment status was a factor in 3 studies (Costa et al., 2022; Perris et al., 2021; Subramaniam et al., 2020). Two studies found that full-time employment, compared to freelance work or unemployment, was associated with treatment delays (Costa et al., 2022; Subramaniam et al., 2020). Conversely, a study of 83 patients with OCD in Italy found that longer DUI was significantly associated with being unemployed (p = 0.028) (Perris et al., 2021).

The factor of gender was observed in 2 studies (Caraveo-Anduaga and Bermúdez, 2004; Stengler-Wenzke et al., 2004). One study found male gender was associated with a delay from symptom onset to diagnosis of 13.2 years for men compared to 8.6 years for women (Stengler-Wenzke et al., 2004). Additionally, men delayed seeking professional help by an average of 10 years, while women delayed for only 6.4 years (Stengler-Wenzke et al., 2004). However, another study found that women often sought assistance from priests and natural healers, while men primarily sought help from mental health specialists (Caraveo-Anduaga and Bermúdez, 2004).

Education was a factor found in 1 study (Zheng et al., 2021). Here, participants in the long DUI (>3 years) group had significantly more years of education (long DUI: 14.30 ± SD2.68, short DUI: 12.57 ± SD3.06, t = −4.24, p < 0.001) (Zheng et al., 2021).

The factor of income was examined in 1 study (Williams et al., 2012). Higher income was positively correlated with being too busy for treatment (r = .251, p = 0.05), but was also correlated with participants having tried treatment (r = .23, p = 0.05) (Williams et al., 2012).

The factor of family history of OCD was identified in 1 study (Viswanath et al., 2011). Compared to those with sporadic OCD, or those who did not have a first-degree relative with OCD, subjects with familial OCD had a longer duration of untreated illness (sporadic: 34.03 ± SD39.78 months and familial: 61.36 ± SD78.09 months) (Viswanath et al., 2011).

#### 3.2.4. Section 2.4: theme 4 - characteristics of OCD

Characteristics of OCD was identified as a theme in 19 studies (Albert, Barbaro, et al., 2019; Belloch et al., 2009; Caraveo-Anduaga and Bermúdez, 2004; Chakraborty et al., 2013; Chong et al., 2012; Del Valle et al., 2018; Del Valle et al., 2017; Dell’Osso et al., 2015; Grant et al., 2007; Karadaĝ et al., 2006; Matsumoto et al., 2021; Okasha et al., 2021; Perris et al., 2021; Poyraz et al., 2015; Stengler et al., 2013; Subramaniam et al., 2020; Vigne et al., 2019; Zheng et al., 2021; Ziegler et al., 2021) and had 5 factors: early age of onset, OCD as compared to other psychiatric disorders, content of obsessions, comorbidities, and symptom characteristics (severity & quality).

Six studies found that an earlier age of onset of symptoms was associated with delays in care (Albert, Barbaro, et al., 2019; Grant et al., 2007; Perris et al., 2021; Poyraz et al., 2015; Stengler et al., 2013; Ziegler et al., 2021). Age of onset is variably defined; in some studies this is the age when OCD symptoms first manifest, and in other studies, this is when OCD symptoms began to cause distress or impairment. One study of 293 participants with OCD found those who experienced symptom onset later in life (over age 29) sought treatment sooner, with an average delay of 5.2 years from symptom onset (Grant et al., 2007). In contrast, those with young adult onset (ages 19–29) and early onset (before age 19) delayed treatment for an average of 7.8 years and 14.7 years, respectively (Grant et al., 2007). Another study found that participants with a longer duration of ASO-AD (age of symptom onset to age of diagnosis) reported symptom onset at an average age of 16.08 years (SD = 8.80), while those in with shorter duration of ASO-AD reported symptom onset on average at 22.85 years (SD = 13.16) (Ziegler et al., 2021).

Six studies in this review compared delays in treatment between OCD and other psychiatric disorders, revealing that patients with OCD had longer treatment and diagnosis delays compared to patients with other psychiatric disorders (which we termed “OCD as compared to other psychiatric disorders” to capture this factor).^10,12,15,16,55,59^ One study compared OCD with other anxiety disorders and found that patients with OCD had a longer DUI than patients with panic disorder (Vigne et al., 2019). Another study found that there were higher odds (88.3 % vs. 73.4 %, Chi-Square test, p = 0.0076) of a treatment gap among those diagnosed with OCD compared to participants with MDD (Subramaniam et al., 2020). Similarly, patients with OCD took significantly longer to recognize symptoms compared to patients with MDD or agoraphobia (Del Valle et al., 2017, 2018).

The content of obsessions was identified as a factor in 5 studies (Albert, Barbaro, et al., 2019; Belloch et al., 2009; Dell’Osso et al., 2015; Karadaĝ et al., 2006; Okasha et al., 2021). Patients in one study endorsed “I felt ashamed by the thought contents” (34.6 %) and “I was afraid (of the thought contents)” (19.2 %) as reasons for delaying seeking treatment (Belloch et al., 2009). Two studies found both religious and sexual obsessions to be associated with delays in care (Karadaĝ et al., 2006; Okasha et al., 2021). In a study conducted in Turkey, participants with religious obsessions or sexual obsessions were found to have longer DUI and longer delays in seeking professional help compared to participants with other obsessions (Karadaĝ et al., 2006). An Egyptian study found both religious and sexual obsessions to be associated with seeking care from a traditional healer (Okasha et al., 2021). Aggressive or harmful obsessions were found to contribute to delays in 1 study (Dell’Osso et al., 2015). The duration of untreated illness was found to be significantly longer in participants with aggressive/checking obsessions (one-way ANOVA: F = 3.58, p < 0.01; F = 3.07, p < 0.01) (Dell’Osso et al., 2015). Contamination obsessions were associated with delays in 1 study (Albert, Barbaro, et al., 2019). Amongst an Italian sample of 251 patients with OCD, those with long DUI (>24 months) had significantly higher subscale scores for contamination obsessions on the Y-BOCS than those with brief DUI (≤24 months) (96 ± SD58.5 vs. 39 ± SD44.8, student t-test, p < 0.001) (Albert, Barbaro, et al., 2019).

Comorbidities was identified as a factor in 4 studies (Albert, Barbaro, et al., 2019; Caraveo-Anduaga and Bermúdez, 2004; Karadaĝ et al., 2006; Matsumoto et al., 2021). In this context, comorbidities refer to the occurrence of an additional mental health disorder alongside the diagnosis of OCD. For instance, one study found that the number of participants with a tic disorder was significantly higher in those with a longer DUI (Matsumoto et al., 2021). Substance use disorder was another condition found to be more prevalent in the longer DUI group, with a study reporting that 18 out of 20 participants in this group had the disorder (Albert, Barbaro, et al., 2019).

Symptom characteristics (severity and quality) as a factor was present in 4 studies (Perris et al., 2021; Poyraz et al., 2015; Zheng et al., 2021; Ziegler et al., 2021). In a study of 100 patients with OCD in Germany, those with longer delays between symptom onset and diagnosis showed significantly more severe symptoms on the Obsessive-Compulsive Inventory Revised (OCI-R) score compared to those with shorter delays (Ziegler et al., 2021). Similarly, in an Italian study, greater severity of OCD symptoms at baseline was associated with longer duration of untreated illness (Perris et al., 2021). Furthermore, in a study of 96 patients with OCD in Turkey, it was found that having symptoms that fluctuated over time was associated with a longer duration of untreated illness (Poyraz et al., 2015).

#### 3.2.5. Section 2.5: theme 5 - logistical barriers

Logistical barriers to treatment were identified in 11 studies, with 3 factors emerging: cost of treatment, lack of access to resources, and travel associated with treatment and care (Chessell et al., 2023; Gentle et al., 2014; Hathorn et al., 2021; Keyes et al., 2018; Kolvenbach et al., 2018; Marques et al., 2010; Matsumoto et al., 2021; Okasha et al., 2021; Poyraz et al., 2015; Vuong et al., 2016; Williams et al., 2012). It is important to note that differences were observed between countries and cultural groups within countries.

The cost of treatment emerged in 8 studies as a barrier (Gentle et al., 2014; Hathorn et al., 2021; Kolvenbach et al., 2018; Marques et al., 2010; Matsumoto et al., 2021; Okasha et al., 2021; Poyraz et al., 2015; Williams et al., 2012). In the Netherlands, where healthcare costs are generally less significant for most individuals, only 1 of 24 participants mentioned discontinuing treatment due to cost (Waumans et al., 2022). Similarly, in Japan, only 3.2 % of participants mentioned financial concerns as a reason to delay care (Matsumoto et al., 2021). In contrast, in Egypt, 24.3 % of participants cited affordability as the reason for preferring traditional healers (Okasha et al., 2021). Even higher proportions of participants in South Africa (72 %) and Singapore (74.6 %) reported financial obstacles to accessing care. In the US, financial concerns and cost of treatment were cited by similar proportions of White (54.6 %) and Black (53.5 %) Americans (Williams et al., 2012).

Lack of access to resources was identified in 7 studies (Chessell et al., 2023; Gentle et al., 2014; Keyes et al., 2018; Kolvenbach et al., 2018; Marques et al., 2010; Vuong et al., 2016; Williams et al., 2012). For instance, in the UK, parents of children with OCD described “challenges, frustration, and helplessness trying to access appropriate support for their child’s OCD” (Chessell et al., 2023). In the Netherlands, long waiting times, problems with the referral process, and difficulty contacting mental health care institutions were noted as significant challenges (Waumans et al., 2022). In the US, 22.2 % of White Americans and 15.5 % of Black Americans were unable to see their preferred provider, and 15.7 % of White Americans and 14.1 % of Black Americans could not secure any appointment (Williams et al., 2012).

Travel associated with treatment and care was a factor in 5 studies (Gentle et al., 2014; Kolvenbach et al., 2018; Marques et al., 2010; Vuong et al., 2016; Williams et al., 2012). A relatively small fraction (3.5 %) of Australians reported they “could not get to treatment because of problems with transportation” (Gentle et al., 2014), while larger portions of individuals in Singapore (20.9 %), the US (25.2 %), and the UK (50 %) reported challenges with travel or transportation contributing to barriers in seeking or receiving care (Kolvenbach et al., 2018; Marques et al., 2010; Subramaniam et al., 2020; Williams et al., 2012).

#### 3.2.6. Section 2.6 - theme 6 - religious and spiritual perspectives

Nine studies highlighted how religious and spiritual beliefs and practices influenced the recognition, treatment, and management of OCD (Al-Solaim & Loewenthal, 2011; Chakraborty et al., 2013; Gentle et al., 2014; Grover et al., 2014; Kolvenbach et al., 2018; Mahintorabi et al., 2017; Matsumoto et al., 2021; Okasha et al., 2021; Poyraz et al., 2015), with 4 factors emerging: seeking religious or spiritual support first, misalignment between religion and mental illness, attributing symptoms to religious or supernatural causes, and the use of religious or spiritual practices and rituals for management of symptoms.

Seeking religious or spiritual support as a first line of treatment or support was identified in 7 studies (Al-Solaim & Loewenthal, 2011; Chakraborty et al., 2013; Grover et al., 2014; Mahintorabi et al., 2017; Matsumoto et al., 2021; Okasha et al., 2021; Poyraz et al., 2015). Many participants mentioned turning to faith-based healers or religious figures in their community for assistance before pursuing medical treatment. For example, 100 % of participants in a study conducted in Saudi Arabia sought a faith-based healer first (Al-Solaim & Loewenthal, 2011), as did 18 % of participants in a study in India (Grover et al., 2014).

Misalignment between religion and recognizing OCD as a mental illness was identified in 6 studies (Al-Solaim & Loewenthal, 2011; Gentle et al., 2014; Grover et al., 2014; Kolvenbach et al., 2018; Mahintorabi et al., 2017; Poyraz et al., 2015). In Saudi Arabia, participants felt that failing to perform religious rituals properly was a punishable sin, and for some, these rituals became compulsions (Al-Solaim & Loewenthal, 2011). In an Australian study, Muslim participants viewed OCD symptoms as part of a religious need for cleanliness, rather than a mental health issue (Mahintorabi et al., 2017). This misalignment is further reflected in beliefs about treatment, with 28 % of participants in India reporting that they expected prayers or rituals to lead to an improvement in symptoms (Grover et al., 2014).

Attributing symptoms to religious or supernatural causes was mentioned in 6 studies (Al-Solaim & Loewenthal, 2011; Chakraborty et al., 2013; Grover et al., 2014; Mahintorabi et al., 2017; Okasha et al., 2021; Poyraz et al., 2015). For example, a study in Turkey found that 15.6 % of participants believed their symptoms were due to being a sinner (Poyraz et al., 2015), while in India, 57.3 % attributed their OCD symptoms to supernatural causes (Grover et al., 2014). In Egypt, of those who preferred traditional healing, 81.1 % associated their symptoms with religion (Okasha et al., 2021).

Finally, the use of religious or spiritual practices and rituals for the management of OCD symptoms was highlighted in 3 studies (Grover et al., 2014; Mahintorabi et al., 2017; Okasha et al., 2021). Individuals are often instructed to engage in additional prayers and religious practices after initially seeing a spiritual advisor for their symptoms; in at least one study, this reportedly exacerbated symptoms of OCD (Mahintorabi et al., 2017). Further, when participants engaged in religious practices as a treatment for OCD, 22 % of participants believed these practices alone would be sufficient to manage their symptoms (Grover et al., 2014).

For clarity, our analysis considers help from traditional healers, faith healers, religious leaders, and other non-clinical sources as "alternative treatments" which take place outside recognized clinical settings and do not qualify as formal "assessment and treatment of obsessive-compulsive disorder." While these sources may provide support, pursuing these forms of help was generally found to be categorized as increasing delays in receiving evidence-based care, reflecting our focus on clinical pathways to diagnosis and treatment.

### 3.3. Section 3: factors associated with decreasing delays in care

Factors motivating participants to seek treatment were identified in 25 studies (Al-Solaim & Loewenthal, 2011; Altamura et al., 2010; Belloch et al., 2009; Benatti et al., 2016; Besiroglu et al., 2004; Caraveo-Anduaga and Bermúdez, 2004; Chakraborty et al., 2013; Chessell et al., 2023; Costa et al., 2022; Cullen et al., 2008; Del Valle et al., 2017; Dell’Osso et al., 2017; Fullana et al., 2009; Gentle et al., 2014; Hathorn et al., 2021; Koran et al., 2000; Mahintorabi et al., 2017; Pedley et al., 2019a; Pedley et al., 2019b; Poyraz et al., 2015; Robinson et al., 2017; Torres et al., 2007; Vigne et al., 2019; Vuong et al., 2016; Williams et al., 2012). The themes of demographic factors and characteristics of OCD contained factors associated both with increases and decreases in delays in care. Four factors were identified that exclusively improved access to care: 1) interference with daily life; 2) motivation from family and friends; 3) positive beliefs and knowledge about OCD, symptoms, or treatment; 4) social burden and relationship strain.

#### 3.3.1. Section 3.1 - theme 1: demographic factors

Demographic factors, including age (Koran et al., 2000), education (Belloch et al., 2009), income (Williams et al., 2012), and family history (Benatti et al., 2016), were associated with reduced delays in 4 studies. One study found that those aged 18–39 years old were more likely to have had treatment for their OCD than those over 40 (Koran et al., 2000). Another study differentiating long and short delays to seeking treatment for OCD found those that sought treatment within a year of diagnosis had a lower educational level (Belloch et al., 2009). A study of Black Americans with OCD found that those who had tried treatment in the past were more likely to have higher incomes (Williams et al., 2012). Another study found that those with family history of psychiatric illness had a significantly younger age at onset and age at first diagnosis (Benatti et al., 2016).

#### 3.3.2. Section 3.2 - theme 2: characteristics of OCD

Characteristics of OCD were associated with decreased delays in 20 studies (Costa et al., 2022; Pedley et al., 2019a; Pedley et al., 2019b; Vigne et al., 2019; Dell’Osso et al., 2017; Del Valle et al., 2017; Mahintorabi et al., 2017; Robinson et al., 2017; Benatti et al., 2016; Vuong et al., 2016; Poyraz et al., 2015; Gentle et al., 2014; Al-Solaim & Loewenthal, 2011; Altamura et al., 2010; Belloch et al., 2009; Fullana et al., 2009; Cullen et al., 2008; Torres et al., 2007; Besiroglu et al., 2004; Caraveo-Anduaga and Bermúdez, 2004) and contained 5 factors: Symptom characteristics (severity & Quality), Comorbidities, Content of obsessions, Early Age of Onset, and OCD as a condition.

Symptom characteristics (severity & quality) were associated with decreased delays in 15 studies (Al-Solaim & Loewenthal, 2011; Belloch et al., 2009; Besiroglu et al., 2004; Cullen et al., 2008; Del Valle et al., 2017; Dell’Osso et al., 2017; Fullana et al., 2009; Gentle et al., 2014; Mahintorabi et al., 2017; Pedley et al., 2019a; Pedley et al., 2019b; Poyraz et al., 2015; Robinson et al., 2017; Vigne et al., 2019; Vuong et al., 2016). One study found that increased symptom severity on the Y-BOCS was positively associated with receiving treatment (Cullen et al., 2008), another study also found that individuals with more severe symptoms had shorter durations of untreated illness (Dell’Osso et al., 2017). Participants also expressed concerns about reaching a breaking point and the potential negative consequences of leaving symptoms unaddressed (Pedley et al., 2019a; Robinson et al., 2017; Vuong et al., 2016).

The presence of comorbidities was identified as decreasing delays in 8 studies (Besiroglu et al., 2004; Caraveo-Anduaga and Bermúdez, 2004; Costa et al., 2022; Cullen et al., 2008; Fullana et al., 2009; Gentle et al., 2014; Torres et al., 2007; Vigne et al., 2019). Comorbid conditions included depressive disorders (Caraveo-Anduaga and Bermúdez, 2004; Fullana et al., 2009), anxiety disorders (generalized anxiety, agoraphobia, social phobia, and hypochondria) (Costa et al., 2022), and mania or hypomania (Cullen et al., 2008), and a study conducted in the UK found that 56 % of individuals with comorbidities sought care compared to only 14 % of those with OCD alone (Torres et al., 2007).

Four studies found that the content of obsessions was correlated with reduced delays (Al-Solaim & Loewenthal, 2011; Besiroglu et al., 2004; Fullana et al., 2009; Poyraz et al., 2015). The absence of contamination/cleaning obsessions was associated with shorter latency to treatment (Costa et al., 2022). Participants with sexual, aggressive, or religious obsessions tended to seek treatment earlier compared to those with contamination, doubt, or control obsessions (Poyraz et al., 2015). In another study, harm-based and shame-based obsessions were associated with earlier help-seeking behavior (Fullana et al., 2009).

While most studies found early age of onset to be associated with longer delays, 3 studies found that an earlier age of onset resulted in earlier treatment seeking (Costa et al., 2022; Dell’Osso et al., 2017; Gentle et al., 2014). Being older than 18 at the time of assessment was associated with longer latency to treatment, with the median latency increasing by 4.4 % for each year above 18 (Costa et al., 2022).

Three studies found that having OCD as a condition motivated individuals to seek treatment sooner compared to other mental health disorders (Altamura et al., 2010; Benatti et al., 2016; Vigne et al., 2019). One study found significant differences in age at first diagnosis, revealing that individuals with OCD were diagnosed earlier than those with generalized anxiety disorder (GAD) (Benatti et al., 2016). In the same study, the age at first treatment for patients with OCD was notably younger compared to GAD patients (Benatti et al., 2016). Additionally, patients with OCD showed the earliest age at first treatment among anxiety disorders (Altamura et al., 2010), and exhibited a shorter duration of untreated illness compared to those with social anxiety disorder (Vigne et al., 2019).

#### 3.3.3. Section 3.3 - Factor 3: motivation from family/friends

Motivation from family and friends emerged as another factor leading to decreased delays in 7 studies (Al-Solaim & Loewenthal, 2011; Belloch et al., 2009; Benatti et al., 2016; Chessell et al., 2023; Del Valle et al., 2017; Mahintorabi et al., 2017; Robinson et al., 2017). Broadly, social support and encouragement from close relationships often prompted individuals to seek professional help sooner.

#### 3.3.4. Section 3.4 - Factor 4: interference with daily life

Interference with daily life was a motivator to seek treatment in 7 studies (Al-Solaim & Loewenthal, 2011; Belloch et al., 2009; Del Valle et al., 2017; Mahintorabi et al., 2017; Pedley et al., 2019a; Robinson et al., 2017; Vuong et al., 2016). Participants often reported that OCD symptoms disrupted their normal productivity and engagement in enjoyable activities, which eventually prompted them to seek professional help.

#### 3.3.5. Section 3.5 - Factor 5: positive beliefs and knowledge about OCD, symptoms, or treatment

Positive beliefs and knowledge about OCD symptoms or treatment were noted in 3 studies as decreasing delays (Chakraborty et al., 2013; Hathorn et al., 2021; Robinson et al., 2017). These included exposure to media and information about OCD, confidence in healthcare professionals, perceived treatment benefits, and personal beliefs about symptoms.

#### 3.3.6. Section 3.6 - Factor 6: social burden and relationship strain

Finally, social burden and relationship strain were also identified as motivators in 2 studies, with OCD symptoms straining relationships and contributing to social impairment (Del Valle et al., 2017; Vuong et al., 2016). This social impact frequently prompted individuals to seek professional intervention.

### 3.4. Section 4: factors not significantly associated with delays

Our scoping review also identified factors that were not significantly associated with delays in seeking diagnosis or treatment. Regarding demographic factors, age (Altamura et al., 2010; Besiroglu et al., 2004; Torres et al., 2007), gender (Besiroglu et al., 2004; Costa et al., 2022; Cullen et al., 2008; Gentle et al., 2014; Grant et al., 2007; Hezel et al., 2022; Levy et al., 2013; Matsumoto et al., 2021; Perris et al., 2021; Poyraz et al., 2015; Torres et al., 2007; Zheng et al., 2021; Ziegler et al., 2021), family history of OCD (Altamura et al., 2010; Dell’Osso et al., 2017; Gentle et al., 2014; Grant et al., 2007; Matsumoto et al., 2021; Perris et al., 2021; Poyraz et al., 2015), marital status (Besiroglu et al., 2004; Costa et al., 2022; Gentle et al., 2014; Perris et al., 2021; Ziegler et al., 2021), education (Besiroglu et al., 2004; Cullen et al., 2008; Gentle et al., 2014; Hezel et al., 2022; Perris et al., 2021; Ziegler et al., 2021), race and ethnicity (Chong et al., 2012; Costa et al., 2022; Fernández de la Cruz et al., 2015; Hezel et al., 2022; Torres et al., 2007), income (Cullen et al., 2008; Gentle et al., 2014; Hezel et al., 2022; Swisher et al., 2023), employment status (Besiroglu et al., 2004; Gentle et al., 2014; Ziegler et al., 2021), and religious affiliation (Gentle et al., 2014; Hezel et al., 2022; Matsumoto et al., 2021), were found not to be associated with delays across a variety of studies. Studies also reported non-significant associations with delays in care: content of obsessions (Dell’Osso et al., 2017; Grant et al., 2007; Levy et al., 2013; Ziegler et al., 2021), comorbidities (Fernández de la Cruz et al., 2015; Hezel et al., 2022; Ziegler et al., 2021), early age of onset (Altamura et al., 2010; Chong et al., 2012; Fernández de la Cruz et al., 2015; Matsumoto et al., 2021), and symptom characteristics (severity, quality, impact) (Fernández de la Cruz et al., 2015; Hezel et al., 2022; Matsumoto et al., 2021; Subramaniam et al., 2020; Torres et al., 2007).

### 3.5. Section 6: trends in factors influencing OCD treatment delays

Our scoping review has covered a substantial body of research exploring factors associated with delays in OCD diagnosis and treatment. Fig. 2 visually summarizes these results, highlighting the number of studies that identified a theme and individual factor as contributing to increased or decreased time to diagnosis and treatment. The three most frequently identified factors associated with increased delays in care were lack of understanding or recognition of OCD symptoms, stigma, and lack of information on OCD. The most frequently identified factors associated with decreased delays in care were symptom characteristics (severity and quality), and comorbidities; motivation from family and friends and interference with daily life were tied as the third most frequently identified factors associated with decreased delays in care.

## 4. Discussion

### 4.1. Overview

Prior research has identified and explored factors that contribute to delays in the assessment and treatment of OCD, and our review aims to evaluate the existing literature in an unbiased manner. We queried multiple databases in July 2023 with broad search terms and screened ~4400 studies, reviewed the full text of 186 studies, and ultimately extracted data from the 51 studies included herein. We used an iterative and inductive process to identify and track factors across all studies and subsequently developed 6 conceptual themes. Our findings identify domains in which improvements to the accessibility of diagnosis and treatment of OCD may be possible, such as through education of the public and providers, policy initiatives, or enhancing support systems for patients and their families.

### 4.2. Principal findings

Our scoping review reveals that a complex interplay of individual, societal, and systemic factors continues to impede timely access to care for individuals with OCD, resulting in prolonged periods of untreated illness that can span years or even decades (Altamura et al., 2010). The findings emphasize the importance of improving early identification and intervention strategies, developing targeted public health initiatives, and implementing more accessible and culturally-responsive treatment approaches, all of which require attention from clinicians, researchers, and policymakers.

First, widespread lack of knowledge and awareness of OCD repeatedly surfaced across studies, with patients believing symptoms were typical experiences and not due to an illness (Del Valle et al., 2017; Gentle et al., 2014; Keyes et al., 2018; Matsumoto et al., 2021; Pedley et al., 2019a), or not related to OCD specifically (Chakraborty et al., 2013; Poyraz et al., 2015; Robinson et al., 2017). Parents also expressed difficulty in distinguishing between normal child development and OCD symptoms (Chessell et al., 2023). Lack of knowledge also extended to healthcare professionals with OCD unrecognized in more than 70 % of cases by consultant psychiatrists (Wahl et al., 2010). However, educational materials and online resources can facilitate connection to quality information and access to care (Bey et al., 2025), and increasing access to content from organizations such as the International OCD Foundation (International OCD Foundation) and related organizations could further improve awareness. Further, more accurate representations of OCD in popular culture, such as in movies and television, could also broaden awareness of this condition.

Perspectives on and behaviors related to care can create barriers to treatment, exposing challenges in healthcare delivery and participant engagement. The relationship between a patient and their healthcare provider is particularly meaningful, and evidence suggests that a better relationship with a general practitioner improves access to treatment (Waumans et al., 2022). Trust in the provider-patient relationship is important and can be influenced by patients’ perception of physician empathy (Wu et al., 2022). Moreover, mistrust is pronounced among ethnic minority groups in multiple countries; in the UK participants report that "cultural differences are not acknowledged in the system" (Kolvenbach et al., 2018) and in the US, Black Americans endorsed statements such as "does not trust White establishment" (Williams et al., 2012). Increasing broad representation of mental healthcare clinicians will allow patients to find providers of similar ethnic and cultural backgrounds, which is likely to improve overall access to care and trust in the physician-patient relationship and the healthcare system more broadly.

Negative perceptions and stigma surrounding OCD also create significant obstacles to treatment-seeking behavior for patients. These perceptions operate on multiple levels – personal, familial, and societal – creating compound barriers to accessing care. For instance, the finding that 30 % of participants delayed treatment due to fear of being labeled "mentally ill" demonstrates stigma’s direct impact on help-seeking behavior (Belloch et al., 2009). Additionally, patients also expressed concern about the potential stigma they would face from their own family regarding a diagnosis (Kolvenbach et al., 2018; Marques et al., 2010; Robinson et al., 2017). Some family members also felt stigmatized by society’s misconceptions of mental illness in general (Stengler-Wenzke et al., 2004). These findings point to the need for interventions that address both public perceptions and individual attitudes toward mental health treatment. The esTOCma app, a gamified intervention aimed at increasing OCD awareness, reducing stigma, and promoting help-seeking, is one potential approach that has shown effectiveness in achieving these outcomes (García-Soriano et al., 2024).

Demographics are associated with disparities in access to care, with age-related barriers consistently documented across studies, and older participants experiencing longer delays from clinical onset to OCD diagnosis (Albert, Barbaro, et al., 2019; Altamura et al., 2010; Chong et al., 2012; Costa et al., 2022; Cullen et al., 2008; Gentle et al., 2014; Hezel et al., 2022; Levy et al., 2013; Matsumoto et al., 2021; Poyraz et al., 2015; Swisher et al., 2023; Zheng et al., 2021; Ziegler et al., 2021). Clinical presentation patterns also influence treatment-seeking behavior, with sexual, and harm-based obsessions associated with delays in care in some studies (Dell’Osso et al., 2015; Karadaĝ et al., 2006; Okasha et al., 2021) and with seeking treatment in other studies (Poyraz et al., 2015). Among anxiety disorders, OCD was found to have the longest duration of untreated illness, averaging 90.57 months (Altamura et al., 2010). The presence of comorbidities shows mixed effects, with some studies showing increased treatment-seeking (Besiroglu et al., 2004; Caraveo-Anduaga and Bermúdez, 2004; Costa et al., 2022; Cullen et al., 2008; Fullana et al., 2009; Gentle et al., 2014; Torres et al., 2007; Vigne et al., 2019), while others found longer delays with certain comorbidities (Albert, Barbaro, et al., 2019; Caraveo-Anduaga and Bermúdez, 2004; Karadaĝ et al., 2006; Matsumoto et al., 2021). These complex patterns suggest that improving early identification and treatment requires a nuanced understanding of how different clinical presentations influence help-seeking behavior. This also suggests that providers should be aware of OCD as a condition across the lifespan and continue to consider this as a new diagnosis in older adults.

Logistical barriers highlight disparities in access to care across healthcare systems. In numerous studies, cost and finances were explicitly noted as a barrier to seeking psychiatric treatment, and these varied across countries (Gentle et al., 2014; Hathorn et al., 2021; Kolvenbach et al., 2018; Marques et al., 2010; Matsumoto et al., 2021; Okasha et al., 2021; Poyraz et al., 2015; Subramaniam et al., 2020; Williams et al., 2012). In Japan, 3.2 % of participants identified financial factors as a barrier (Matsumoto et al., 2021), while in South Africa, this figure rose to 72 % (Hathorn et al., 2021). Relatedly, lack of insurance coverage emerged as a systemic barrier, with participants in multiple studies reporting their insurance did not cover OCD treatment (Gentle et al., 2014; Marques et al., 2010; Williams et al., 2012). Transportation barriers disproportionately affect certain populations, with 37 % of Black Americans reporting transportation difficulties compared to 23 % of White Americans in the US. (Williams et al., 2012) Solutions to these barriers should include fair coverage of treatment for OCD by insurance providers and improved access to cost effective care, potentially through the use of online and digital resources, known to be an effective treatment option (Wu et al., 2023; Feusner et al., 2022; Hiranandani et al., 2023)

Finally, religious and spiritual factors influence treatment-seeking patterns. Studies included herein found religious and spiritual factors to be pronounced in Islamic societies and across the Middle East, where traditional and religious approaches to mental health diagnosis and treatment are frequently used before or in place of clinical care. The findings that 81.1 % of participants in some regions preferred traditional healing due to religious factors (Okasha et al., 2021), and that 57.3 % attributed their symptoms to supernatural causes (Grover et al., 2014), highlight the complex interaction between spiritual beliefs and healthcare utilization. Religious leaders were often seen by participants as better equipped to handle symptoms, particularly those with religious content (Al-Solaim & Loewenthal, 2011). The observation that religious practices sometimes exacerbated symptoms (Mahintorabi et al., 2017) points to a critical need for collaboration between mental health professionals and religious leaders. Those who practice psychiatry should be aware of the role religion and spirituality play in their patients’ lives and engage with them accordingly. Resources that directly speak to this interplay between OCD, spirituality, and faith can provide information both for patients as well as providers (International OCD Foundation). Training psychiatry residents to take spiritual histories, discussing case studies with chaplains, and reflecting on personal biases or blind spots can improve patient engagement and enhance culturally competent care (Awaad et al., 2015).

In contrast to factors associated with delays in diagnosis and treatment, several factors emerged as significant motivators for treatment-seeking behavior, potentially offering insights into strategies for reducing treatment delays. Social support, particularly from family and friends, was identified as a frequent catalyst for seeking treatment (Al-Solaim & Loewenthal, 2011; Belloch et al., 2009; Benatti et al., 2016; Chessell et al., 2023; Del Valle et al., 2017; Mahintorabi et al., 2017; Robinson et al., 2017). The impact of symptoms on daily functioning served as another motivator, with disruption to productivity and enjoyable activities prompting help-seeking behavior (Al-Solaim & Loewenthal, 2011; Belloch et al., 2009; Del Valle et al., 2017; Mahintorabi et al., 2017; Pedley et al., 2019a; Robinson et al., 2017; Vuong et al., 2016). Positive beliefs and knowledge about OCD symptoms or treatment emerged as another facilitating factor, with studies highlighting how exposure to OCD through the media, confidence in healthcare professionals, and understanding of treatment benefits motivated help-seeking behavior (Chakraborty et al., 2013; Hathorn et al., 2021; Robinson et al., 2017). This finding reinforces recommendations for increased representation of OCD in popular culture as a means to improve understanding and access to care.

Collectively, these findings indicate that delays in diagnosis and treatment of OCD result from a complex interaction of individual, social, and systemic factors that current global healthcare systems are not effectively addressing. The evidence suggests that successful intervention requires a multi-level approach that simultaneously targets knowledge gaps, attitudes towards mental health care, stigma, healthcare delivery, and practical barriers to access. Particularly concerning are the disparities in access and treatment delays among different demographic groups, highlighting the need for targeted interventions that address the unique barriers faced by specific populations. The identification of factors that facilitate treatment-seeking, such as family support and symptom recognition, provides insights for developing effective early intervention strategies. Future research should focus on evaluating integrated interventions that can address multiple barriers simultaneously, with particular attention to cultural competency and accessibility. These efforts should be guided by the understanding that reducing treatment delays requires both removing obstacles to care and strengthening the factors that motivate and enable treatment-seeking behavior. Ultimately, meaningful progress in reducing delays in OCD diagnosis and treatment will depend on collaborative, inclusive approaches that prioritize patient voices and adapt continuously to the evolving needs of diverse communities.

### 4.3. Strengths & limitations

This scoping review offers a comprehensive overview of factors associated with delays in assessment and treatment of OCD, offering an important update since the last review, published in 2021, which focused on 21 studies conducted in the US. (Senter et al., 2021) The systematic approach ensured that all relevant studies were included, and the blinded screening and data extraction by study team members allowed for a more objective reporting of findings.

Our study may be limited by exclusion of non-English language studies. However, this study criteria resulted in only 7 studies being excluded from our scoping review, with 2 being in Spanish, 2 in German, 2 in Turkish, and 1 in Mandarin. Further, given that the initial search was done between July 20, 2023, and July 27, 2023, studies published after this period were not included and may have offered additional insights into our research question. Additionally, we included studies that demonstrated indirect relationships between a number of factors relating to delays in assessment and treatment of OCD, potentially limiting our ability to draw causal relationships between these factors. Nonetheless, we feel that including these indirect factors provides the broadest overview of the challenges in timely diagnosis and treatment of OCD. Finally, our analytic approach of developing themes of factors by necessity simplifies the multifaceted nature of the factors that contribute to delays in assessment and treatment of OCD so that connections between studies could be explored. Thus, it is important to acknowledge that individuals with OCD often face nuanced and intersecting barriers to care that extend beyond the categories we have established here. Further research should prioritize understanding these complex, interconnected barriers to OCD diagnosis and treatment while developing targeted solutions that can effectively address both individual-level obstacles and systemic challenges across diverse populations and healthcare settings.

## Figures and Tables

**Table 1:** Factors Associated with Delays in Assessment and Treatment of Obsessive-Compulsive Disorder. Summary of studies examining misunderstanding OCD, perspectives and behaviors surrounding care, demographic factors, characteristics of OCD, logistical barriers, and religious and spiritual perspectives. #Study design is qualitative or mixed methods. *Multiple Mental Disorders Discussed in Studies. BACE: Barriers to Access Care Evaluation; CSM: Common-Sense Model of Self-Regulation; HBM: Health Belief Model; IASMHS: Inventory of Attitudes Toward Seeking Mental Health Services; IP: Illness Perceptions; IPQ-R: Illness Perception Questionnaire-Revised; AD: Age Diagnosed; ASO: Age of Symptom Onset; ASO-AD: Age of Symptom Onset to Age of Diagnosis; BTQ: Barriers to Treatment Questionnaire; CIS-R: Clinical Interview Schedule-Revised; DI: Duration of illness; DUI: Duration of untreated illness; FOCI-B: Florida Obsessive-Compulsive Inventory; GAF: Global Assessment of Functioning; HDRS: Hamilton Depression Rating Scale; LTT: Latency to treatment; NHS: National Health Service; OCD-IRS: Obsessive-Compulsive Disorder Intrusiveness Rating Scale; Q-LES-Q-SF: Quality of Life Enjoyment and Satisfaction Questionnaire; USS: Universal Stigma Scale.

Author (Year; Country)	Population Description	Aim	Themes & Factors	Main Outcomes
Chessell et al. (2023; United Kingdom) (Chessell et al., 2023) #	Parents of preadolescent children (7- to 12-years-old) with OCD. Parents were recruited from two Child and Adolescent Mental Health Services (CAMHS) in Southeast England, UK-based mental health charities, social media, and UK-based private treatment providers.	“This study therefore conducted in-depth, semi-structured qualitative interviews to build on the existing literature and explore experiences of parenting a preadolescent child (7- to 12-years-old) with OCD.”	Factors associated with increased delays: Misunderstanding OCD: Lack of understanding or recognition of OCD symptoms Lack of information on OCD Perspectives and behaviors surrounding care: Denial Logistical barriers: Lack of access to resources Factors associated with decreased delays: Motivation from family/friends	Parents found it difficult to identify OCD-related symptoms or distinguish from other mental health disorders, hormonal changes, or normative childhood development. Parents found it difficult coming to terms with the diagnosis, not using family accommodation, and getting help for the child, but after accepting their child’s OCD, they were able to better support their child and seek appropriate help. Parents faced long waiting times and were unable to access therapists/healthcare providers. Parents wanted to help their child but did not know how.
Swisher et al. (2023; United States) (Swisher et al., 2023)	Participants with self-reported OCD were recruited through clinician Web sites and treatment sites, social media platforms (e.g., Instagram), and OCD advocacy Websites (e.g., International OCD Foundation).	“The present study examined the relationship of family functioning with obsessive-compulsive symptom severity, depression severity, and duration of treatment delay in a sizable sample of adults with self-reported obsessive-compulsive symptoms.”	Factors associated with increased delays: Demographic factors: Age Race and ethnicity Marital status Factors not significantly associated with delays: Demographic factors: Income	Age shows a strong positive correlation (R^2^ = .51) with treatment delay (p < 0.001). White participants have significantly longer treatment delays (Mean = 7.94 years, SD = 9.12) compared to non-White participants (Mean = 3.92 years, SD = 4.71), t(87) = 3.53, p < 0.001 Married participants have significantly longer treatment delays (Mean = 11.35 years, SD = 12.16) compared to single participants (Mean = 5.55 years, SD = 5.97), t(59) = 3.22, p < 0.01. Those who had longer delays in seeking treatment were more likely to be older and have higher income (p = 0.15).
Costa et al. (2022; Brazil) (Costa et al., 2022)	Patients with OCD included in the 1001-person outpatient sample from the Brazilian Research Consortium on Obsessive-Compulsive Spectrum Disorders (CTOC).	“In this study, we aimed to estimate the LTT (latency to treatment) and to assess its de-mographic and clinical correlates among well characterized OCD pa-tients who have sought treatment at some point along the course of their illness."	Factors associated with increased delays: Demographic factors: Age Employment Factors associated with decreased delays: Characteristics of OCD: Comorbidities Early age of onset Content of obsessions Factors not significantly associated with delays: Demographic factors: Gender Race and ethnicity Marital status	1/3 of OCD patients took up to 2 years to initiate treatment for OCD, after becoming distressed or impaired by their OCD symptom; 1/3: 2–9 years; 1/3: 10 or more years. Age older than 18 at the time of the assessment was associated with a longer latency to treatment (LTT (p < 0.001). Median LTT increased 4.4 % for each year >18. Factors associated with shorter LTT: Free-lance working conditions associated with 45.6 % lower median LTT than full-time employment (Median Ratio (MR) = .544, p = 0.033). Hypochondriasis (MR = .492, p = 0.011). Absence of symptoms of contamination/cleaning (MR = .687, p = 0.012). Presence of symptoms of the aggression dimension (MR = .744, p = 0.039). There is no statistically significant difference in latency to treatment based on marital status (p = 0.557), ethnicity (p = 0.167) and gender (p = 0.561).
Hezel et al. (2022; United States) (Hezel et al., 2022)	Adults who contacted an OCD outpatient research clinic associated with an academic medical center and who met DSM-5 criteria for a principal diagnosis of OCD	"The aim of this study was to determine whether the delay between OCD onset and diagnosis has decreased in the past 25 years. We also explored how factors such as socioeconomic status, race, and age were associated with time to diagnosis."	Factors associated with increased delays: Demographic factors: Age Factors not significantly associated with delays: Demographic factors: Gender Race and ethnicity Education Income Religious affiliation Characteristics of OCD: Comorbidities Symptom characteristics (severity & quality)	Significant mean delay of 11.4 years (SD = 8.9) between OCD symptom onset and OCD diagnosis by a clinician (t (159) = −16.1, p < 0.001) and 7.1 years (SD = 8.7) between clinical OCD onset and OCD diagnosis (t(164) = −10.5, p < 0.001). Years to diagnosis was positively correlated with age, with older participants experiencing a greater delay from clinical onset to OCD diagnosis (F (1,163) = 18.3, p < 0.001). No significant differences in mean number of years from clinical OCD onset to diagnosis when comparing men and women, racial and ethnic background, income, or religious affiliation A bivariate correlation revealed no significant associations between number of years from clinical onset to diagnosis and years of education, current OCD severity, number of current or lifetime comorbid diagnoses, or quality of life (all ps ≥.44)
Waumans et al. (2022; Netherlands) (Waumans et al., 2022) * #	Outpatients with generalized anxiety disorder, social anxiety disorder, panic disorder, OCD, PTSD, and/or MDD from an outpatient department for mental health care in the Netherlands.	“The aim of this study was to examine both barriers and facilitating factors of treatment-seeking in patients with anxiety or depressive disorders in a Western-European healthcare setting, using qualitative methods.”	Factors associated with increased delays: Misunderstanding OCD: Lack of understanding or recognition of OCD symptoms Perspectives and behaviors surrounding care: Stigma	Participant with OCD did not recognize her symptoms of OCD, despite being familiar with the psychiatric classification system. Another participant who developed OCD during pregnancy considered her psychological complaints without an understandable cause abnormal, which in combination with ridiculing statements about her symptoms from her environment made her feel ashamed, contributing to stigma as a hindrance to treatment seeking.
Hathorn et al. (2021; South Africa) (Hathorn et al., 2021)	Adults with OCD living in South Africa who were recruited from an existing database and completed the online survey.	“We aimed to (1) investigate the role of the health belief model (HBM) constructs in predicting help-seeking intention among a group of South African adults with OCD, and (2) to determine the most endorsed barriers to help-seeking for OCD in this sample.”	Factors associated with increased delays: Perspectives and behaviors surrounding care: Stigma Denial Doubts on effectiveness of treatments Logistical barriers: Cost of treatment Factors associated with decreased delays: Positive beliefs and knowledge about OCD, symptoms, or treatment	4 most endorsed barriers (Barriers to Access Care Evaluation): Wanting to solve the problem on my own (90 %) Not being able to afford the financial costs involved (72 %) Feeling embarrassed or ashamed (66 %) Concerns about the treatments available (e.g., medication side effects) (66 %) Perceived treatment benefits was the only predictor variable that significantly predicted help-seeking intention [B = 1.37, t(42) = 5.16, and p < 0.01]
Ferreira et al. (2021; Portugal) (Ferreira et al., 2021) #	33-year-old male with pedophilia-themed OCD brought to the emergency department after presenting with suicidal ideation.	“Our aim is to present a case report highlighting the role stigma plays in delaying treatment, the clinical challenges in the diagnosis, and in the treatment of pedophilia-themed OCD, in order to address the lack of literature on the subject.”	Factors associated with increased delays: Misunderstanding OCD: Lack of understanding or recognition of OCD symptoms Perspectives and behaviors surrounding care: Stigma	Believed that even staring at his nephews’ photos could make him a pedophile Avoidance of triggers led to isolation. Overall, low insight into his condition. Patient experienced strong stigma toward psychiatric treatment
Matsumoto et al. (2021; Japan) (Matsumoto et al., 2021)	New outpatients who visited the OCD specialty outpatient clinic of Kyoto Prefectural University of Medicine (KPUM) between June 1, 2017 and May 31, 2019.	“Differences in medical environments have been reported to affect the DUI. Therefore, we surveyed the DUI of OCD in Japan and the reason for delayed treatment."	Factors associated with increased delays: Misunderstanding OCD: Lack of understanding or recognition of OCD symptoms Perspectives and behaviors surrounding care: Stigma Mistrust in health professionals Denial Fear Seeking alternative treatments Not wanting to take medication Logistical barriers: Cost of treatment Religious and spiritual perspectives: Seeking religious or spiritual support first Demographic factors: Age Characteristics of OCD: Comorbidities (only tic disorders) Factors not significantly associated with delays: Demographic factors: Gender Family history Religious affiliation Characteristics of OCD: Early age of onset Symptom characteristics (severity & quality)	Reasons for delaying the first hospital visit: Did not think OCD symptoms were associated with an illness (64.5 %); Belief could manage OCD symptoms (58.1 %); Not disturbed by symptoms (38.7 %); Ashamed of symptoms and needing help (29.0 %); Possibility of medication use (26.7 %); Spontaneous fluctuation of symptoms (19.4 %); Symptoms as necessary to be tidy (19.4 %); Afraid of a diagnosis of mental illness (16.1 %); Did not want to discuss OCD symptoms with psychiatrist (16.1 %); Perception that treatment will be ineffective (12.9 %); Family support for overcoming symptoms (6.5 %); Feeling depressed (3.2 %); Financial factors (3.2 %); Prefer to go to other department doctors or spiritual healer (3.2 %) The members of the DUI >2 years group were significantly older (Wilcoxon rank sum test, p < 0.01). The proportion of patients with a history of tic disorders was significantly higher in the DUI >2 years group (chi-square test, p < 0.01). There were no significant differences in sex, age at onset of OCD, Y-BOCS scores, OCI scores, BDI-II scores, and the comorbidities of depression and autism spectrum disorder (ASD) or attention-deficit/hyperactivity disorder (ADHD) between the groups. There was no significant difference in family history of psychiatric disorders (p = 0.21) or family history of OCD (p = 0.59)
Okasha et al. (2021; Egypt) (Okasha et al., 2021)	Adult OCD patients seeking treatment at the inpatient ward or outpatient clinic of the Okasha Institute of Psychiatry, Ain Shams University.	"This study is a part of our aforementioned project (Okasha, 2004) that aimed to assess the role of traditional healers among the pathway of psychiatric services of OCD patients. We aimed also to highlight the relationship between the type of symptomatology and other clinical variables and the help seeking behavior among traditional healers."	Factors associated with increased delays: Misunderstanding OCD: Lack of understanding or recognition of OCD symptoms Perspectives and behaviors surrounding care: Stigma Logistical barriers: Cost of treatment Religious and spiritual perspectives: Seeking religious or spiritual support first Attributing symptoms to religious or supernatural causes Use of religious or spiritual practices and rituals for management of symptoms Characteristics of OCD: Content of obsessions	39.8 % went to traditional healers, and 94.6 % were before psychiatric advice. 40.6 % went to traditional healers within the first year of illness, 24.4 % within the first five years of illness (p = 0.011). 81.1 % of those receiving care from traditional healer associated OCD with religion, 45.9 % considered symptoms to be related to magic or superstitions. Reasons for seeing a traditional healer: Stigma surrounding psychiatric advice (89.2 %); Family members recommendation (81.1 %); Affordability (24.3 %) Traditional healers use practices that are culture-specific and based on community beliefs. Significant associations with seeking traditional healers’ advice: presence of sexual obsessions (*χ*2 = 4.646, p = 0.031) and religious obsessions (*χ*2 = 8.244, p = 0.004).
Perris et al. (2021; Italy) (Perris et al., 2021)	Outpatients with OCD from the Department of Psychiatry of the University of Campania Luigi Vanvitelli, in Naples, Italy.	"This is a real-world, naturalistic, follow-up study evaluating the impact of DUI on long-term clinical outcomes. The main hy-pothesis of this longitudinal study is that DUI is influenced by several socio-demographical factors and that DUI, insight and severity of OCD symptoms are the main predictors of patients’ longitudinal positive remission, considered as outcome in our study."	Factors associated with increased delays: Demographic factors: Employment Characteristics of OCD: Early age of onset Symptom characteristics (severity & quality) Factors not significantly associated with delays: Demographic factors: Gender Marital status Family history Education	DUI was longer in patients who were unemployed (OR = 2.780, p = 0.028), with an earlier onset of the disorder (OR = −.145, p = 0.042) and a greater severity of OCD symptoms at baseline (OR = .280, p = 0.008). Linear regression models were used to identify clinical and socio-demographic variables associated with DUI, including gender (p = 0.793), age at onset (p = 0.037), marital status (p = 0.269), family history of OCD (p = 0.340), employment status (p = 0.028), and education (p = 0.708); with only early age at onset and employment being statistically significant.
Ziegler et al. (2021; Germany) (Ziegler et al., 2021)	OCD patients currently undergoing outpatient treatment from the Department of Psychiatry and Psychotherapy at the University of Leipzig Medical Center in Germany.	"This study aimed to investigate the duration between symptom onset and diagnosis (duration ASO-AD) and the duration between diagnosis and beginning of a therapy (duration AD-therapy) in a sample of patients undergoing outpatient treatment for OCD. Further, differences between patients with a short duration ASO-AD and a long duration ASO-AD regarding various sociodemographic and clinical variables were analyzed."	Factors associated with increased delays: Demographic factors: Age Characteristics of OCD: Early age of onset Symptom characteristics (severity & quality) Factors not significantly associated with delays: Demographic factors: Gender Marital status Education Employment Characteristics of OCD: Content of obsessions Comorbidities	Average age of symptom onset (ASO) = 18.72 years (SD = 11.24); average age diagnosed (AD) = 31.71 years (SD = 12.57) The mean duration ASO-AD = 12.78 years (SD = 11.30). The mean duration AD to start of therapy = 1.45 years (SD = 4.51). In the group with short duration (< 7 years) ASO-AD, duration ASO-AD ranged from 0 to 5 years; in the group with a long duration (≥7 years) ASO-AD, duration ASO-AD ranged from 7 to 45 years. The two subgroups differed significantly in: age, ASO, AD, duration ASO-AD, OCI-R total score, OCI-R ordering and neutralizing subscales, GAF, and self-stigmatization (USS); all p < 0.05. The effects were large for duration ASO-AD, medium for ASO, AD and age, and small for the remaining variables Patients with a short duration ASO-AD were significantly younger (p = 0.001) but reported average ASO = 22.85 years (SD = 13.16), whereas patients with a long duration ASO-AD reported average ASO = 16.08 years (SD = 8.80) (p < 0.001). Patients with a long duration ASO-AD had significantly more severe symptoms (p = 0.035), especially in the subcategories ordering (p = 0.025) and neutralizing (p = 0.046). Short duration ASO-AD showed significantly higher functioning levels (p = 0.016) and less self-stigmatization (p = 0.006). Groups showed no significant differences in gender, marital status, educational level, employment status, presence of children, type of symptoms (checking, hoarding, etc.), independent and media use to search for information regarding treatment, and the number of psychiatric comorbidities
Zheng et al. (2021; China) (Zheng et al., 2021)	OCD outpatients from a specialty clinic (consecutive admissions) from 2017 to 2020.	"This study examined the relationship between response rate and DUI in a sample of individuals with primary OCD who completed an open-label, 12-month SRI follow up treatment."	Factors associated with increased delays: Demographic factors: Age Marital status Education Characteristics of OCD: Symptom characteristics (severity & quality) Factors not significantly associated with delays: Demographic factors: Gender	56.5 % had not been treated before. The mean age for first adequate pharmacological treatment was 23.64 (±8.54) years, and the whole sample showed a mean DUI of 4.07 (±3.49) years. The two sub-samples, long DUI and short DUI (≤3 years, based on median DUI), had significant differences in age (t = −4.78, p < 0.001), education years (t = −4.24, p < 0.001), age of first treatment (t = −3.83, p < 0.001), Y-BOCS scores at baseline (t = −1.99, p = 0.047), and marital status (*χ*2 = 8.74, df = 1, p = 0.003). The average duration of illness was significantly prolonged in the group with a long DUI (>3 years) (10.30 ± 6.87 vs 3.10 ± 3.81; z = −10.37, p < 0.001) There was no statistically significant difference in gender (*X*^2^ = 1.15; p = 0.282)
Subramaniam et al. (2020; Singapore) (Subramaniam et al., 2020) *	Singapore citizens and permanent residents who participated in the Singapore Mental Health Study in 2016 (Shafie et al., 2021)	"The aims of the current study were to establish the 12-month treatment gap and its associated factors among adults with mental disorders in the Singapore resident population using data from the second Singapore Mental Health Study and to examine the changes since the last mental health survey which was conducted in 2010.”	Factors associated with increased delays: Demographic factors: Employment Characteristics of OCD: OCD as a condition Factors not significantly associated with delays: Characteristics of OCD: Symptom characteristics (severity & quality)	Patients with OCD had the 2nd highest treatment gap, 84 % (percentage of those who needed but were not receiving treatment) Univariate analyses of all conditions showed significantly higher treatment gap for those employed as compared (81.9 % vs. 56.9 % unemployed, p = 0.036) and those with mild symptoms (83.5 % vs. 67.1 % moderate/severe symptoms, p = 0.0155). For OCD specifically, no significant difference in treatment gap between mild and moderate symptoms (86.4 % vs. 13.6 %, Chi-Square test, p = 0.087). There were significantly higher odds of treatment gap among those diagnosed with OCD compared to those with MDD (88.3 % vs. 73.4 %, Chi-Square test, p = 0.0076)
Albert et al. (2019a; Italy) (Albert et al., 2019a)	Adult patients (≥18 years of age) with a principal OCD diagnosis and Y-BOCS total score ≥16 who were referred to the Department by San Luigi Gonzaga University Hospital in the years 1998–2017.	"1. estimate the mean duration of untreated illness in a large sample of individuals with OCD; and 2. to investigate its impact on response to the first ever adequate pharmacological treatment."	Factors associated with increased delays: Demographic factors: Age Marital status Characteristics of OCD: Content of obsessions Comorbidities Early age of onset	A mean DUI of 106.19 ± 118.14 months was calculated, and 65 % of patients reported a long DUI (>24 months). Between the Long DUI (>24 months) and brief DUI (≤24 months), there was a significant difference in age (40.41 ± 13.78 vs. 31.61 ± 11.32, student t-test, p < 0.001) and marital status (of those in brief DUI 71.3 % were single vs 52.4 % in long DUI, chi-square test, p < 0.05). Compared to the brief DUI group, the long DUI group had a younger age of symptoms onset (15.76 ± 7.57 years vs. 17.92 ± 8.04 years, student t-test, p < 0.001) and younger age of disorder onset (21.14 ± 9.23 years vs. 24.22 ± 8.83 years, student t-test, p < 0.001). Compared to the brief DUI group, the long DUI group had a higher score for the contamination obsessions subscale of the Y-BOCS checklist (96 ± 58.5 vs. 39 ± 44.8, student t-test, p < 0.001). Compared to the brief DUI group, the long DUI group had a greater number of patients with substance use disorders (18 vs. 2, student t-test, p < 0.001).
Pedley et al. (2019a; United Kingdom) (Pedley et al., 2019a) #	Individuals with OCD aged 16 or older who were recruited by open advertisement or who were participants in a multi-site OCD treatment trial (‘OCTET’).	"This study aims to use qualitative methods to identify and characterize the dimensions of IP in OCD. The findings will make it possible to evaluate the utility of the CSM in OCD and to ascertain whether any adaptation of the current model and its associated measures is needed."	Factors associated with increased delays: Misunderstanding OCD: Lack of understanding or recognition of OCD symptoms Lack of information on OCD Perspectives and behaviors surrounding care: Mistrust in health professionals Doubts on effectiveness of treatments Not wanting to take medication Factors associated with decreased delays: Interference with daily life Characteristics of OCD: Symptom characteristics (severity & quality)	Took many years to recognize OCD, and some perceived behaviors as “normal” or consistent with their personality, or attributed experiences to other mental health disorders. Delayed therapy as it ‘was not the right time to receive it.’ Concerns about effectiveness due to variability in therapist quality or difficulty with continuity given staff ‘turnover’. Experienced shame, causing them to conceal their OCD from health professionals. Doubtful about the effectiveness of medication as a standalone treatment and felt it did not address the underlying problem. Failure to recognize OCD led to an increase in symptom severity, prompting treatment to control and improve their OCD symptoms.
Pedley et al. (2019b; United Kingdom) (Pedley et al., 2019b)	People aged 16 or older with a self-reported diagnosis of OCD from a health professional, recruited through advertisement and direct invitation.	"The primary aim of this study was to test the psychometrics of a new version of the IPQ-R, which has been adapted for OCD following preliminary qualitative work. A secondary aim was to examine associations between perceptions of OCD with emotional responses, help-seeking intentions and treatment use."	Factors associated with decreased delays: Characteristics of OCD: Symptom characteristics (severity & quality)	Those who saw OCD as more permanent were significantly more likely to plan to seek NHS help in the future (p = 0.01) Those who had received talk therapy perceived significantly more control (IPQ-R ‘control’ sub-scale) over their OCD. Participants who had a more coherent understanding of their OCD scored higher (more positive attitudes) on IASMHS sub-scales psychological openness (*τ* = .18) and indifference to stigma (*τ* = .24)
Vigne et al. (2019; Brazil) (Vigne et al., 2019) *	Patients who sought treatment at the anxiety and obsessive-compulsive disorders Clinic of the Institute of Psychiatry of the Federal University of Rio de Janeiro (IPUD/UFRJ) and who had a main diagnosis of OCD, panic disorder (PD), or social anxiety disorder (SAD).	“In this study, we compared DUI using the first contact with a mental health professional in OCD, PD, and SAD patients and investigated its correlates, both within specific diagnoses and across the whole sample.”	Factors associated with increased delays: Characteristics of OCD: OCD as a condition Factors associated with decreased delays: Characteristics of OCD: Comorbidities Symptom characteristics (severity & quality) OCD as a condition	DUI correlated negatively with the perception of OCD being caused by stress (ρ = −.39, p = 0.03) The DUI was significantly different between groups that sought treatment after the onset of illness (*χ*^2^ = 20.5; df = 2; p < 0.001), with patient with OCD having longer DUI (7.73 [10.51] years) than patients with PD (1.46 [2.62] years: *χ*^2^ = 8.99; df = 1; p = 0.003) and shorter DUI than patients with SAD (13.72 [12.72] years; *χ*^2^ = 4.12; df = 1; p = 0.04).
Del Valle et al. (2018; Spain) (Del Valle et al., 2018)*	Adult ambulatory patients with a diagnosis of OCD, agoraphobia, major depressive disorder, anorexia nervosa, or cocaine dependence in mental health outpatient clinics of the NHS.	“To obtain information about the time that patients with various mental disorders take to (a) recognize that they have a problem (disorder awareness), (b) disclose the problem and/or the difficulties in managing the problem to a third party and (c) seek specialist professional help.”	Factors associated with increased delays: Perspectives and behaviors surrounding care: Stigma Fear Characteristics of OCD: OCD as a condition	Patients with OCD, agoraphobia, major depressive disorder, anorexia nervosa, and cocaine dependence reported similar levels of fear and stigma as barriers to seeking help Patients with AGO and MDD took significantly less time to recognize their symptoms than other groups (F = 3.382, p < 0.01. The OCD, AN, and COC patients took longer to disclose their problems with no significant differences between them (F = 2.105, .p < 0.05). None of the groups differed in factor 3 (i.e. motivators for not seeking help: fear and stigma), which indicates that there is a similar level of personal stigma and fear of social rejection, irrespective of the disease suffered.
Kolvenbach et al. (2018; United Kingdom) (Kolvenbach et al., 2018) #	A convenience sample of parents from White and ethnic backgrounds of children and adolescents [who had been assessed in the clinic and were either on the waitlist to receive treatment for OCD or had already started specialist treatment] were recruited from the National and Specialist OCD, BDD, and Related Disorders Clinic for Young People, South London and Maudsley NHS Foundational Trust, London, United Kingdom.	“To describe the barriers that parents from different ethnic groups identify when accessing specialist services for OCD and investigate whether these are different across ethnic backgrounds.”	Factors associated with increased delays: Misunderstanding OCD: Lack of understanding or recognition of OCD symptoms Lack of information on OCD Perspectives and behaviors surrounding care: Stigma Mistrust in health professionals Denial Fear Doubts on effectiveness of treatments Logistical barriers: Cost of treatment Lack of access to resources Travel associated with treatment and care Religious and spiritual perspectives: Misalignment between religion and mental illness Demographic factors: Race and Ethnicity	Endorsed by both White (W) and ethnic minority (EM) parents as barriers: Lack of knowledge or education about mental health issues (W n = 9, EM n = 10) Lack of resources in public health system: (W n = 8, EM n = 6) Negative previous experiences in public health system (W n = 8, EM n = 5) Lack of trust in the mental health system (treatment, confidentiality, recording) (W n = 6, EM n = 7) Time (W n = 5, EM n = 6) Money (W n = 5, EM n = 4) Lack of emotional and practical support outside the public health system (W n = 4, EM n = 3) Bullying (W n = 4, EM n = 4) Inconvenient location (W n = 1, EM n = 2) Endorsed mostly by EM parents: Stigma and discrimination in family and community (W n = 2, EM n = 9) Shame/denial (W n = 0, EM n = 6) Lack of trust in mental health system (general) (W n = 1, EM n = 7) Different cultural beliefs about mental health issues (W n = 0, EM n = 5) Endorsed mostly by ethnic minority (EM) (vs white (W)) parents as barriers: Stigma and discrimination in family and community (W n = 2, EM n = 9): Lack of support and stigma in extended family; Stigma in community or cultural group Shame/denial (W n = 0, EM n = 6): Embarrassment; Not willing to admit there is a problem Lack of trust in mental health system (W n = 1, EM n = 7): Cultural group mistrusts the mental health system Different cultural beliefs about mental health issues (W n = 0, EM n = 5): Different cultural or religious beliefs about mental illness; Cultural differences are not acknowledged in the system Discrimination within system (W n = 0, EM n = 2): Systematic discrimination due to minority status
Keyes et al. (2018; United Kingdom) (Keyes et al., 2018) #	14–17-year-old adolescents with OCD recruited through three Tier 3 Child and Adolescent Mental Health Services (CAMHS) in the United Kingdom.	“The current study therefore investigates the issues faced by young people leading up to the development of OCD-related difficulties, and explores their experiences of living with the challenges of obsessions and compulsions”	Factors associated with increased delays: Misunderstanding OCD: Lack of understanding or recognition of OCD symptoms Perspectives and behaviors surrounding care: Stigma Mistrust in health professionals Denial Doubts on effectiveness of treatments Logistical barriers: Lack of access to resources	Participants struggled to understand their experiences, often keeping them secret and worrying they were "going crazy." Believed their behaviors were normal, contributing to feelings of being misunderstood or different from others. Ambivalence with seeking help: mental health interventions not always timely or helpful. Therapy, particularly Exposure and Response Prevention (ERP), was often perceived as challenging, with some participants experiencing resistance or conflict. Long waitlists for mental health services caused frustration and delayed access to care.
Dell’Osso et al. (2017; Italy) (Dell’Osso et al., 2017)	Consecutive OCD outpatients were recruited over a period of 10 years from the University Hospital Policlinico, OCD Unit, in Milan, Italy. This is an increased study sample including patients from Dell’Osso et al., 2015 (Dell’Osso et al., 2015).	“The present study was aimed to further examine clinical and demographic features associated with increased severity of illness, defined through standardized psychometric scales and evaluated in a categorical perspective, in an Italian tertiary clinic-sample of OCD patients.”	Factors associated with decreased delays: Demographic factors: Age Characteristics of OCD: Early age of onset Symptom characteristics (severity & quality) Factors not significantly associated with delays: Demographic factors: Family history Characteristics of OCD: Content of obsessions	The group with an increased severity of illness was found to be younger (mean age 38.6 ± 16.3 vs. 43.8 ± 13.6 years, p < 0.05) and showed earlier age at onset (21.3 ± 10.5 vs. 27.5 ± 14.1 years, p < 0.01) and age at first pharmacological treatment (28.9 ± 12.1 vs. 36.1 ± 15.1, p < 0.01) (Student’s t-test: t = 1.8, p < 0.05; t = 2.7, p < 0.01; p < 0.01, respectively) In the group with increased severity, the duration of illness was found to be longer (Student’s t-test: t = .48, p < 0.01), while the DUI shorter (Student’s t-test: t = .38, p < 0.01) in the increased severity group. No significant differences between the two groups were found in terms of family history of psychiatric disorders, including OCD and the presence of obsessive/compulsive symptomatology.
Del Valle et al. (2017; Spain) (Del Valle et al., 2017)*	Adult ambulatory patients with a diagnosis of OCD, agoraphobia, major depressive disorder, anorexia nervosa, or cocaine dependence in mental health outpatient clinics of the NHS.	"The aims of this study were, first, to examine the whole process of seeking help for mental health problems using a structured interview, and second, to compare this process in five groups of individuals with different mental disorders that have a high impact on healthcare resources: OCD, Agoraphobia (AGO), Major Depressive Disorder (MDD), Anorexia Nervosa (AN), and Cocaine Dependence (COC)."	Factors associated with increased delays: Misunderstanding OCD: Lack of understanding or recognition of OCD symptoms Lack of information on OCD Perspectives and behaviors surrounding care: Stigma Mistrust in health professionals Denial Fear Characteristics of OCD: OCD as a condition Factors associated with decreased delays: Interference with daily life Motivation from family/friends Social burden and relationship strain Characteristics of OCD: Symptom characteristics (severity & quality)	Barriers: The thought “the symptoms will not last (63.6 %); Can keep symptoms under control (54.5 %); Shame (50.9 %); Give little importance to symptoms (45.5 %); Fear of being considered crazy (43.6 %); Thought that “It’s not dangerous” (39.1 %); Fear of social rejection (29.4 %); Guilt about the symptoms (20.6 %); Low interference by symptoms (17.3 %); Low disturbance by symptoms (16.2 %); Don’t know who to tell (14.7 %); Fear of treatment (11.8 %); Mistrust in health professionals (11.8 %); Low frequency of symptoms (8.8 %); Nobody to trust (5.9 %); Thought-Action Fusion beliefs (5.5 %); Thought that “It’s common” (or usual) (4.5 %) Reasons for recognizing a problem: Anxiety/distress increases (78.6 %); Symptoms’ uncontrollability (75.3 %); Greater interference in daily life (71.6 %); Disturbing behavior changes (59.5 %); Symptom frequency increases (59.5 %); Sadness increases (50.0 %); Someone said it wasn’t normal (35.1 %) Motivators of help seeking: Anxiety (81.4 %); Out of control (77.1 %); Interference in daily life (71.4 %); It didn’t go away (71.4 %); Frequency of symptoms increases (68.6 %); Fear of the symptoms increasing (62.5 %); It had control over my life (62.1 %); Fear (59.6 %); Sadness (57.1 %); Fear of being crazy (47.7 %); Thought-Action Fusion (45.7 %); Thought that it was something bad (43.5 %); Someone tells me get help (40.6 %); My health is in danger (30.0 %); Being a bad person (18.6 %) Patients with OCD took significantly longer to recognize symptoms compared to AGO and MDD (27.26 (SD = 62.39) vs 3.76 (SD = 11.45) and 4.56 (SD = 13.22) months respectively, F4,147 = 3.382, p < 0.05, ηp2 = .082) Patients with OCD had significantly longer disclosure times than MDD (40.60 (SD = 67.91) vs 5.83 (SD = 13.29) months, F4,147 = 2.105, p < 0.05, ηp2 = .067) Patients with OCD had significantly longer help-seeking times than MDD (53.01 (SD = 83.36) vs 8.00 (SD = 13.50) months, F4,147 = 2.240, p < 0.05, ηp2 = .055) Higher guilt about symptoms in OCD than AGO (20.6 % vs 6.7 %, *χ*2 = 13.32, p < 0.01) Lower ability to keep symptoms under control in OCD than MDD and AN (54.5 % vs 93.8 % and 91.7 %, *χ*2 = 23.76, p < 0.001) Lower rates of viewing symptoms as "common" in OCD than AN and COC (4.5 % vs 33.3 % and 59.1 %, *χ*2 = 20.66, p < 0.001) Patients with OCD had significantly different age of onset than AN and MDD (24.59 (SD = 9.95) vs 17.92 (SD = 3.01) and 41.50 (SD = 10.45) years, F4,148 = 17.67, p < 0.001, ηp2 = .322) Patients with OCD had significantly different duration of disorder than AGO and AN (9.58 (SD = 9.75) vs 7 (SD = 7.93) and 6.54 (SD = 5.79) years, F4,148 = 3.87, p < 0.05, ηp2 = .094)
Mahintorabi et al. (2017; Australia) (Mahintorabi et al., 2017) #	Muslim adult women with OCD washing subtype.	“The present study sought to explore treatment seeking among practicing Muslim women living with OCD-washing subtype] in Australia.”	Factors associated with increased delays: Misunderstanding OCD: Lack of understanding or recognition of OCD symptoms Lack of information on OCD Perspectives and behaviors surrounding care: Denial Doubts on effectiveness of treatments Religious and spiritual perspectives: Seeking religious or spiritual support first Misalignment between religion and mental illness Attributing symptoms to religious or supernatural causes Use of religious or spiritual practices and rituals for management of symptoms Factors associated with decreased delays: Interference with daily life Motivation from family/friends Characteristics of OCD: Symptom characteristics (severity & quality)	Religious OCD symptoms as causing the most interference with life activities and leading reason for seeking treatment. OCD symptoms as a function of religious need for cleanliness/pureness, not as a mental health disorder. Some participants felt that religion both provided support and exacerbated their OCD symptoms. Some may have been hesitant to admit to negative effects caused by religion because of fear of punishment/blasphemy. One participant did not want to seek help at all, until she realized that her symptoms were impacting her family members, and generally, the last option. First approach to seeking help was to speak to an Imam, and some of the advice from the Imams was to engage further in the prayers and OCD rituals. In two cases, participants mentioned that the medications they had taken were not helpful in decreasing their OCD symptoms, and they still suffered from religious OCD symptoms.
Robinson et al. (2017; United Kingdom) (Robinson et al., 2017) #	People with an official diagnosis and those who self-identified as having OCD who were contacted through OCD-UK, a charity run by people with personal experience of OCD.	"The present study sought to address this need for qualitative data through in-depth, individual interviews, conducted by a researcher with personal experience of OCD. The objective of the study was to identify the factors that encourage people with OCD to seek help and the reasons why people do not seek treatment."	Factors associated with increased delays: Misunderstanding OCD: Lack of understanding or recognition of OCD symptoms Lack of information on OCD Perspectives and behaviors surrounding care: Stigma Mistrust in health professionals Denial Fear Factors associated with decreased delays: Interference with daily life Motivation from family/friends Positive beliefs and knowledge about OCD, symptoms, or treatment Characteristics of OCD: Symptom characteristics (severity & quality)	Barriers: Stigma: stigma in different contexts including not wanting to tell people, not wanting to tell the doctor, fear about having OCD on their medical record, wanting to shield the family, or family not wanting to acknowledge participant having an illness. Internal/Cognitive Factors: OCD was not bad enough, could manage on their own, reluctant to accept there may be a problem, never thought of getting help, did not deserve treatment, and felt too vulnerable. Not knowing what it was Factors related to GP/Treatment: concerns if GP would know what the problem was, how GP would react, and general concerns. Fear of Criminalization Enablers: Being supported or urged to seek treatment: encouraged by family Crisis/Crunch Point: reached a breaking point Media/Information: learned about OCD through media or educational resources. Confidence in GP/Mental Health Professionals Driven to seek treatment because of the nature of the thoughts
Benatti et al. (2016; Italy) (Benatti et al., 2016) *	Consecutive in-patients and out-patients, diagnosed with OCD, PD, or GAD, attending the psychiatric services of the University Clinics of Milano, Monza-Brianza, and Cagliari	“The present study aimed to explore the influence of sociodemographic and clinical factors in relation to onset and latency to treatment in patients with generalized anxiety disorder (GAD), panic disorder (PD), and obsessive–compulsive disorder (OCD).”	Factors associated with decreased delays: Demographic factors: Family History Motivation from family/friends Characteristics of OCD: OCD as a condition	For age at first diagnosis, significant differences were observed when comparing GAD with OCD (41.6 ± 15.9 vs. 28.07 ± 11.07 years; F = 13.0, Bonferroni post-hoc: p < 0.001). For age at first treatment, significant differences were observed when comparing GAD versus OCD (43.01 ± 16.3 vs. 27.9 ± 10.9 years, F = 15.4, p < 0.001). Significant differences were found between groups in “autonomous” versus “driven by others” treatment-seeking decisions: PD: 61.3 vs. 38.7 %, GAD: 60.3 vs. 39.7 %, OCD: 32.5 vs. 67.5 %; *χ*2 = 9.5, p < 0.05) It was found that patients with a family history of psychiatric illness had a significantly younger age at onset and age at first diagnosis (respectively: t = 3.4, P < 0.001; t = 3.3, P < 0.001)
Vuong et al. (2016; United Kingdom) (Vuong et al., 2016) #	A convenience sample of participants recruited by an advertisement on a national OCD charity website, website forums, Facebook, Twitter, and newsletters; there was no guarantee that participants had an official diagnosis of OCD.	"The study aimed to explore the process of help-seeking in people experiencing OCD symptoms in order to: (1) identify factors that contribute to recognizing one’s OCD; (2) identify patterns of help-seeking for OCD; (3) gain an understanding about the barriers and enablers faced during help-seeking."	Factors associated with increased delays: Misunderstanding OCD: Lack of understanding or recognition of OCD symptoms Lack of information on OCD Misdiagnoses Perspectives and behaviors surrounding care: Stigma Mistrust in health professionals Fear Doubts on effectiveness of treatments Seeking alternative treatments Logistical barriers: Lack of access to resources Travel associated with treatment and care Factors associated with decreased delays: Interference with daily life Social burden and relationship strain Characteristics of OCD: Symptom characteristics (severity & quality)	Themes from survey: Awareness and understanding: lack of understanding by health professionals, their GP, public and family, causing them not to discuss their difficulties, or being referred to inappropriate treatment. Health care system: problems with the help-seeking process such as long wait times, misdiagnosis, and lack of understanding, which deterred desire to seek help or prompt treatment seeking outside of NHS. Participants did not want to be led down an ‘incorrect treatment path.’ CBT: CBT, especially in a group setting, was not positive; may have helped understanding of OCD but did not cure it. Information: Limited availability of information on OCD, specifically about the lesser known symptoms of OCD. Impetus to seek help: patient (57.5 %); parents during their younger years (17.2 %) or adulthood (5.7 %); a health professional (2.3 %); friends (1.1 %); school (1.1 %) Factors affecting decision to seek help: It became harder to cope with everyday life’; ‘it becoming harder to cope with work’; ‘it affecting relationships.’ Deterrents to seek help: shame and embarrassment (76.1 %); fear of talking about problem (63.6 %); fear of hospitalization (29.5 %); worry about the treatment (29.5 %); worry about how it would disrupt life (21.6 %) or affect work (19.3 %). 54.2 % found receiving initial help was inadequate.
Dell’Osso et al. (2015; Italy) (Dell’Osso et al., 2015)	Consecutive adult outpatients with OCD in an outpatient clinic at the University department of Psychiatry at Fondazione IRCCS Ca’Granda, Ospedale Maggiore Policlinico.	“Few studies have investigated whether clinical phenotypes differ in terms of latency to treatment (i.e., duration of untreated illness [DUI], duration, and severity of illness. The present study was aimed to quantify the aforementioned variables in a sample of OCD patients.”	Factors associated with increased delays: Characteristics of OCD: Content of obsessions	DUI and DI were found to be significantly longer in the aggressive/checking subgroup when compared to the other subgroups (one-way ANOVA: F = 3.58, p < 0.01; F = 3.07, p < 0.01) and this aggressive/checking subgroup had significantly higher Y-BOCS scores (27.4 (±15.37)). No other significant differences among subgroups in terms of DUI and DI were found A strong, positive correlation between DUI and DI (r = .71, n = 114, p = 0.00) was found in the whole sample, with a longer DUI associated with a long DI. The coefficient of determination R2 resulted in 50 % of shared variance.
Fernandez de la Cruz et al. (2015; United Kingdom) (Fernández de la Cruz et al., 2015)	White and non-White youth OCD patients consecutively referred to and treated at the National and Specialist OCD and Related Disorders Team at the Maudsley Hospital, London.	"To compare the clinical characteristics of a sample of White vs. non-White children and adolescents with OCD treated at a national specialist clinic in the UK.”	Factors not significantly associated with delays: Demographic factors: Race and ethnicity Characteristics of OCD: Comorbidities Early age of onset Symptom characteristics (severity & quality)	There were no statistically significant differences in terms of age at assessment, age of onset of OCD, presence of comorbid disorders, or phenomenology of OCD symptoms between White and non-White individuals.
Poyraz et al. (2015; Turkey) (Poyraz et al., 2015)	Consecutive outpatients with OCD who attended the Department of Psychiatry of Cerrahpaşa Medical School, University of Istanbul, Turkey, between September 2013 and September 2014.	“The aim of the present study was to identify potential variables associated with delays in seeking treatment among patients with OCD."	Factors associated with increased delays: Misunderstanding OCD: Lack of understanding or recognition of OCD symptoms Lack of information on OCD Perspectives and behaviors surrounding care: Stigma Mistrust in health professionals Denial Fear Doubts on effectiveness of treatments Seeking alternative treatments Not wanting to take medication Logistical barriers: Cost of treatment Religious and spiritual perspectives: Seeking religious or spiritual support first Misalignment between religion and mental illness Attributing symptoms to religious or supernatural causes Demographic factors: Age Characteristics of OCD: Early age of onset Symptom characteristics (severity & quality) Factors associated with decreased delays: Characteristics of OCD: Content of obsessions Symptom characteristics (severity & quality) Factors not significantly associated with delays: Demographic factors: Gender Family history	Reasons for not seeking treatment: Symptoms were fluctuating spontaneously (61.5 %); Thinking symptoms were not associated with an illness (60.4 %); Belief that one could manage or handle symptoms on his/her own (55.2 %); Not being significantly disturbed by symptoms (33.3 %); Possibility of using medication (24 %); Ashamed of symptoms and needing help (21.9 %); Thinking that symptoms are necessary in order to be tidy/orderly (17.7 %); Feeling depressed/hopeless (15.6 %); Preferring to go to a neurologist/psychologist or spiritual healer (15.6 %); Thinking that symptoms are related to religious problems/being a sinner (15.6 %); Perception that treatment will be ineffective (14.6 %); Afraid to have a diagnosis of mental illness (12.5 %); Logistic or financial factors (12.5 %); Not comfortable discussing OCD related symptoms with the psychiatrist (8.3 %); Not starting treatment even after seeing a psychiatrist (6.3 %) Among patients with poor insight the possibility of using medication (p = 0.03) was significantly associated with a longer DUI. The subgroup with a DUI >4 years was significantly older (Mann-Whitney U, z = −2.191, p = 0.02). Age of OCD onset was significantly lower in the DUI >4 (Mann-Whitney U, z = −4.023, p < 0.001) Believing that OCD symptoms were not associated with an illness was significantly associated with a longer DUI (Chi-square test, p = 0.039). DUI was significantly shorter among patients with a faster onset due to a major precipitating condition compared with patients with insidious onset without a precipitating life event (Chi-square test, p = 0.002) Among patients with poor insight, symptoms fluctuating over time was significantly associated with a longer DUI (Chi-square test, p = 0.047). Patients with sexual/aggressive/religious obsessions showed a trend toward seeking treatment earlier compared with patients with contamination/doubt/control obsessions (Mann-Whitney *U* test, z = −1.703, p = 0.089). DUI subgroups did not significantly differ on gender (*χ*2 = .071, df = 1, p = 0.79), latest pharmacological treatment prescribed (p = 0.393), and family history for OCD (p = 0.792)
Grover et al. (2014; India) (Grover et al., 2014)	Patients with OCD 15 years or older attending the outpatient clinic of the Department of Psychiatry of a tertiary care hospital in north India (Postgraduate Institute of Medical Education and Research (PGIMER), Chandigarh) during the period of July 2011 to December 2011.	“The aim of this article is [to] study the supernatural beliefs and pathways of care in patients with OCD attending the outpatient clinic of a tertiary care center. Additionally, an attempt was made to study the relationship between supernatural beliefs and the first treatment contact.”	Factors associated with increased delays: Misunderstanding OCD: Lack of understanding or recognition of OCD symptoms Lack of information on OCD Religious and spiritual perspectives: Seeking religious or spiritual support first Misalignment between religion and mental illness Attributing symptoms to religious or supernatural causes Use of religious or spiritual practices and rituals for management of symptoms	65.2 % first contacted a psychiatrist; 18 % first contacted faith healers and other traditional healers; 16.9 % first contacted a physician (including the registered medical practitioners) 57.3 % attributed symptoms to a supernatural cause: Planetary influences (37 %), bad deeds in previous life (33 %), divine wrath (24 %), sorcery/witchcraft (19 %), spirit intrusion (18 %), evil spirits (13 %), ghosts (13 %) Beliefs about social and biological causes: Chemical imbalance (55 %), Stress (52 %), One of the two (68 %) Beliefs/patterns on treatment: Performed prayers or other religious rituals with the expectation that it will lead to improvement (28 %); Sought magico-religious help (26 %); Only prayers can lead to improvement (22 %); Only performance of magico-religious rituals (*Jhad-Phook*) can lead to improvement (7 %)
Gentle et al. (2014; Australia) (Gentle et al., 2014)	Adults who identified as having OCD/OCD symptoms, recruited through online advertisements placed on OCD support group websites, university clinic websites, OCD social networking sites, and print advertisements placed in psychology clinics and provided to OCD support groups in the Sydney area.	“The present study aimed to identify the most commonly reported barriers to seeking treatment among Australians who self-reported that they experienced OCD symptoms … The study also sought to determine the proportion of the sample who had received a diagnosis by a health professional, and to examine whether there were differences between those who had received a diagnosis of OCD and those who had not, in demographic characteristics, severity of self-reported symptoms, quality of life, symptom intrusiveness, religious affiliation and identified barriers to treatment. Relationships between specific treatment barriers and religious affiliation were also explored … A final aim of the study was to obtain information about the types of treatment being received by Australians with OCD symptomatology and the level of satisfaction with these treatments.”	Factors associated with increased delays: Misunderstanding OCD: Lack of understanding or recognition of OCD symptoms Lack of information on OCD Perspectives and behaviors surrounding care: Stigma Mistrust in health professionals Denial Fear Doubts on effectiveness of treatments Logistical barriers: Cost of treatment Lack of access to resources Travel associated with treatment and care Religious and spiritual perspectives: Misalignment between religion and mental illness Demographic factors: Race and ethnicity Factors associated with decreased delays: Characteristics of OCD: Comorbidities Early age of onset Symptom characteristics (severity & quality) Factors not significantly associated with delays: Demographic factors: Gender Marital status Family history Education Employment Income Religious affiliation	“I wanted to handle my problems on my own” ranked by 43.1 % as extremely influential in their decision to delay or avoid OCD treatment Barriers ranked as *very much or extremely important*: Embarrassment about the OCD (37.2 %); Lack of motivation (30.2 %); Cost of treatment (29.1 %); Embarrassment about needing help (27.9 %); Lack of information about available services (27.9 %); Concerns about being judged or criticized (27.9 %) Additional barriers (percentage of the group that rated each barrier as *very much or extremely important*): I was afraid treatment would be too upsetting (24.5 %); I was worried about being judged or criticized by my family if I sought treatment (23.3 %); I felt like my symptoms were normal for someone in my situation (22.1 %); I was afraid of being committed to a hospital against my will (22.1 %); I received treatment before and it did not help with my problems (21 %); My health insurance does not cover treatment (20.9 %); I did not think treatment could help with my problems (19.8 %); There is no time in my schedule for treatment (12.8 %.); I could not choose the person I wanted to see for treatment (12.8 %); I don’t trust mental health professionals (11.6 %); I was not satisfied with the treatments that were available (10.5 %); I could not get an appointment (10.4 %); I could not get to treatment because of problems with transportation (3.5 %); I could not find a mental health professional of my same race or ethnicity (2.3 %) Greater religious attendance was correlated with a higher likelihood of identifying cultural factors as a barrier to OCD treatment (p = 0.020). Organized religious attendance was also associated with: lack of knowledge of services, preference for handling problems alone, and belief that the symptoms were ‘normal’ 2.3 % rated the barrier "I could not find a mental health professional of my same race or ethnicity" as very much or extremely important. For the barrier “I was afraid of being treated badly in treatment because of my race or ethnicity”, 2.3 % rated it as their second rank barrier, and 2.3 % rated it as their third rank barrier. Differences between professional diagnosis group and without professional diagnosis: Comorbidity rates - higher in professional diagnosis (84.2 %) than those without (62.1 %). Mean age of first symptoms - earlier in professional diagnosis than those without (13.95 years, SD = 9.3 vs 16.21 years, SD = 11.2). Severity of OCD symptoms - significantly higher in professional diagnosis compared to those without (16.53 vs 12.79, p < 0.05) Q-LES-Q-SF- significantly lower in professional diagnosis compared to those without (37.22 vs 41.65, p < 0.05) OCD Intrusiveness Rating Scale Score - significantly higher in professional diagnosis compared to those without (53.89 vs 37.14, p < 0.05) There was no significant relationship between having an OCD diagnosis and gender, *χ*2(1, N = 86) = .05, p = 0.83, relationship status, *χ*2(6, N = 86) = 3.81, p = 0.70, religious affiliation, *χ*2(4, N = 86) = 3.51, p = 0.48, level of education, *χ*2(6, N = 86) = 6.11, p = 0.41, employment, *χ*2(4, N = 86) = 6.43, p = 0.17, region of origin, *χ*2(6, N = 86) = 4.22, p = 0.65, annual income *χ*2(8, N = 86) = 5.92, p = 0.66, or residing in a capital city *χ*2(2, N = 86) = 2.15, p = 0.34
Chakraborty et al. (2013; India) (Chakraborty et al., 2013) *	120 consecutive adult patients with schizophrenia or related psychotic disorders; mood disorders; OCD and other anxiety disorders; somatization and dissociative disorders attending the outpatient clinic of a tertiary care hospital in India.	“This index study aims to explore the explanatory model of illness and patterns of help seeking behavior from the families of patients with four categories of psychiatric disorders viz. schizophrenia and related psychotic disorders; mood disorders; OCD and other anxiety disorders; somatization and dissociative disorders attending the outpatient of a tertiary care hospital in Eastern India."	Factors associated with increased delays: Misunderstanding OCD: Lack of understanding or recognition of OCD symptoms Lack of information on OCD Religious and spiritual perspectives: Seeking religious or spiritual support first Attributing symptoms to religious or supernatural causes Characteristics of OCD: OCD as a condition Factors associated with decreased delays: Positive beliefs and knowledge about OCD, symptoms, or treatment	80 % of respondents with OCD and anxiety disorders viewed the problem arising out of too much worrying/thinking. 40 % of respondents with OCD thought OCD was due to a kind of medical illness, malfunction/dysfunction of a specific organ system or organ, or black magic and witchcraft. 20 % of patients thought OCD was due to masturbation, spirit, or harm caused by an envious neighbor All the families of patients with OCD and anxiety disorders took recourse to non-professional medical help before coming to the tertiary center, whereas more than half of the families each in the psychotic disorder (67.8 %), depressive disorder (54.6 %) and somatization & dissociative disorder (53.9 %) group sought such treatment. A belief in having some bodily pathology prompted relatives to seek professional medical help for the patient (Spearman’s rank correlation 1.0, p < 0.01), but belief in psychological and supernatural causation drove them toward religious remedies (Spearman’s rank correlation 1.0, p < 0.01).
Levy et al. (2013; United States) (Levy et al., 2013)	Individuals who contacted The Center for the Treatment and Study of Anxiety (CTSA) at the University of Pennsylvania to inquire about treatment for OCD.	“We had two aims: first, to describe the characteristics of treatment-seeking individuals with OCD; and second, to compare the characteristics of individuals who initiated treatment and those who did not.”	Factors associated with increased delays: Demographic factors: Age Factors not significantly associated with delays: Demographic factors: Gender Characteristics of OCD: Content of obsessions	Average age of individuals in the intake group (who had scheduled an intake evaluation) was younger than individuals in the phone screen-only groups (t(1, 352) = 2.05, p = 0.041) Neither obsession nor compulsion type significantly differed between groups. Gender did not significantly differ between groups.
Stengler et al. (2013; Germany) (Stengler et al., 2013)	OCD patients who had come to the University Hospital Outpatient Clinic in Leipzig, Germany, wishing to receive a medical diagnosis and therapy.	“Several studies have described the deficits in the health care provided to persons with OCD, however, without making any distinction between psychiatric–psychotherapeutic professionals and general practitioners or other professionals. Also, the relation between subjectively defined early signs of the disorder, diagnosis and utilization of professional help has not yet been investigated systematically. The present study addresses these questions, using a self-rating questionnaire for patients with OCD.”	Factors associated with increased delays: Demographic factors: Gender (male) Characteristics of OCD: Early age of onset	The mean delay between first occurrence of symptoms and seeking professional help was 8 years. Men delayed seeking professional help on average by 10 years, while women sought professional help on average after 6.4 years The average delay from symptom onset to OCD diagnosis was 10.8 years. The delay from symptom onset to OCD diagnosis for men was 13.2 years; for women, it was 8.6 years. The delay in seeking professional help for early onset (before age 17) was 11.7 years, while the delay in seeking professional help for late onset (after age 17) was 5.6 years; the difference of 6.1 years was statistically significant
Chong et al. (2012; Singapore) (Chong et al., 2012) *	Singapore citizens and permanent residents who participated in the Singapore Mental Health Study in 2010 (Subramaniam, 2012) (Subramaniam et al., 2012).	"This paper reports on the treatment gap of adults with mental disorders in the Singapore resident population, and also presents the projected cumulative lifetime probability of treatment contact and associated factors for each of these disorders."	Factors associated with increased delays: Demographic factors: Age Characteristics of OCD: OCD as a condition Factors not significantly associated with delays: Demographic factors: Race and ethnicity Characteristics of OCD: Early age of onset	Treatment gap (percentage of individuals who require care but don’t receive it) highest in Alcohol abuse (96.2 %), followed by OCD (89.8 %). The lowest proportion of treatment contact seen in those with OCD (18.1 %). Younger cohort had higher odds of making treatment contact for MDD, dysthymia, GAD, and OCD (age group: 18–34 OR = 4.2, (1.5–12.1 95 % CI)). Ethnicity was not associated with treatment contact in OCD. Earlier age at onset was not associated with treatment contact in OCD.
Williams et al. (2012; United States) (Williams et al., 2012) #	Black Americans with a current diagnosis of OCD recruited (over a nine-month period during 2009–2010) by the CTSA at the University of Pennsylvania. A White American subset of an OCD Internet sample was collected by Marques et al. (2010).	"The purpose of this study is to determine what barriers prevent Black Americans from receiving treatment for OCD through a comprehensive assessment of symptoms and self-reported concerns about treatment issues."	Factors associated with increased delays: Misunderstanding OCD: Lack of understanding or recognition of OCD symptoms Lack of information on OCD Perspectives and behaviors surrounding care: Stigma Mistrust in health professionals Denial Fear Doubts on effectiveness of treatments Logistical barriers: Cost of treatment Lack of access to resources Travel associated with treatment and care Demographic factors: Race and ethnicity Income Factors associated with decreased delays: Demographic factors: Income	47.1 % of participants endorsed not realizing they had a disorder or treatment was available; Insurance status negatively correlated with cost concerns (point-biserial correlation, r = −.387, p < 0.01) Barriers endorsed: Felt ashamed of needing help (White American (WA) = 57.41 %, Black American (BA) = 56.34 %) Wanted to handle it on own (WA = 51.85 %, BA = 64.79 %) Worried about cost (WA = 54.63 %, BA = 53.52 %) Felt ashamed of problem (WA = 50.93 %, BA = 47.89 %) Didn’t think treatment would work (WA = 44.44 %, BA = 49.30 %) Health insurance would not cover (WA = 41.67 %, BA = 46.48 %) Worried about what people would think (WA = 38.89 %, BA = 50.70 %) Afraid of criticism by family if sought psychiatric help (WA = 36.11 %, BA = 40.85 %) Too inconvenient or will take too much time (WA = 25.93 %, BA = 38.03 %) Uncomfortable discussing problems with professional (WA = 25.93 %, BA = 38.03 %) Problems with transportation or scheduling (WA = 23.15 %, BA = 36.62 %) Scared about being involuntarily hospitalized (WA = 23.15 %, BA = 29.58 %) Not satisfied with services that were available (WA = 27.78 %, BA = 18.31 %) Could not choose provider (WA = 22.22 %, BA = 15.49 %) Could not get an appointment (WA = 15.74 %, BA = 14.08 %) Communication concerns because of language barriers (WA = 3.70 %, BA = 8.45 %) Statistically significant barriers: Received treatment before that didn’t work (White American (WA) = 38.89 %, Black American (BA) = 12.68 %) Fisher’s Exact Test, p = 0.000 Unsure about who to go see or where to go (WA = 50.00 %, BA = 76.06 %) Fisher’s Exact Test, p = 0.001 Would be treated unfairly because of race or ethnicity (WA = 7.41 %, BA = 22.54 %) Fisher’s Exact Test, p = 0.006 BA participants who had tried treatment before tended to have a higher income (r = .23, p = 0.05); no correlation for education nor insurance status. Pearson and point-biserial correlational analyses of the Mokken scales with demographic variables revealed that income was positively correlated with being too busy for treatment (Pearson correlation, r = .251, p < 0.05)
Al-Solaim and Loewenthal (2011; Saudi Arabia) (Al-Solaim & Loewenthal, 2011) #	Muslim females between the ages of 14 and 30 (with an onset of OCD before the age of 21) attending psychiatric clinics in Saudi Arabia.	“To explore the role of religion in the experience of OCD among young, female, Saudi sufferers attending psychiatric clinics in Saudi Arabia."	Factors associated with increased delays: Misunderstanding OCD: Lack of understanding or recognition of OCD symptoms Lack of information on OCD Perspectives and behaviors surrounding care: Mistrust in health professionals Doubts on effectiveness of treatments Fear Religious and spiritual perspectives: Seeking religious or spiritual support first Misalignment between religion and mental illness Attributing symptoms to religious or supernatural causes Factors associated with decreased delays: Interference with daily life Characteristics of OCD: Symptom characteristics (severity & quality) Motivation from family/friends	All interviewees reported their first source for treatment was a faith-based healer, and this was an appropriate outlet for help; psychiatry service was perceived as the last resort. On average 6–12 months elapsed between contact with a faith-based healer and contact with a mental health professional (MHP). Patients went to an MHP after acknowledging that benefits of faith healing were limited for OCD symptoms. As young women, the interviewees did not have agency to seek help themselves, and decisions on treatment seeking involved family. Patients accepted the biological aspect of the mental illness but believed that obsessional symptoms were due to the evil eye and metaphysical power. Patients felt that a religious professional would be more conscientious and would not harm nor manipulate them, would better understand religious symptoms, and would be more knowledgeable of the religious view of certain behaviors, allowing them to give better advice. Failure to carry out religious rituals in the proper manner was seen as a punishable sin. Patients found that symptoms related to daily prayers and rituals were the most distressing, time-consuming, and interfering with their normal lives, and the main reason for seeking treatment.
Viswanath et al. (2011; India) (Viswanath et al., 2011)	Familial and sporadic OCD patients from the National Institute of Mental Health and Neurosciences, Bangalore, India.	“In this study, we reviewed the clinical records of familial and sporadic OCD patients to compare their clinical characteristics, comorbidity and treatment response.”	Factors associated with increased delays: Demographic factors: Family history	DUI was significantly longer in familial compared to sporadic OCD groups (t = 2.84, p = 0.005, 61.36 ± 78.09 vs 34.03 ± 39.78 months) Familial OCD groups had significantly: Earlier age of onset (t = −2.41, p = 0.017, 20.33 ± 10.05 vs 24.15 ± 10.22 years) Higher baseline YBOCS compulsions scores (t = 2.39, p = 0.018, 11.66 ± 4.94 vs 9.51 ± 6.15) Higher rates of comorbidity (chi-square test, 75.0 % vs 48.8 %, p = 0.001) Higher rates of mixed OCD (both obsessions and compulsions) (Fisher’s exact test, 86.9 % vs 75.0 %, p = 0.04)
Altamura et al. (2010; Italy) (Altamura et al., 2010) *	Consecutive outpatients diagnosed with MDD, bipolar disorder type I and II (BDI and BDII), GAD, PD, and OCD, attending the Mood Disorder Unit within the Department of Psychiatry, University of Milan.	“The purpose of this study was to investigate and compare age at onset, age at first treatment and DUI in patients with different mood and anxiety disorders”	Factors associated with decreased delays: Characteristics of OCD: OCD as a condition Factors not significantly associated with delays: Demographic factors: Age Family history Characteristics of OCD: Early age of onset	With respect to the age at first treatment in patients with anxiety disorders (F = 17.405, P = 0.001), patients with OCD showed the earliest age at first treatment (33.22, SD = 12.28), but no statistical difference was found between patients with OCD and PD. Amongst patients with anxiety disorders (F = 12.076, P < 0.0001), patients with OCD had overall the earliest age of onset (25.78, SD = 12.18; OCD vs. GAD p < 0.0001; OCD vs. PD p = 0.02). Amongst patients with anxiety disorders (F = 7.512; P < 0.00), patients with OCD showed the longest DUI (90.57 months, SD = 106.11), but no statistical difference was found between patients with OCD and GAD. Patients with OCD had more frequently a positive family history for psychiatric disorders compared with the other groups, whereas patients with PD had less frequently a positive family history for psychiatric disorders (w2 = 26.263, P < 0.0001)
Marques et al. (2010; United States) (Marques et al., 2010)	Adults recruited through advertisements placed in online OCD forums, on OCD clinic websites around the Boston area with following criteria: (1) complete data for the Yale–Brown Obsessive Compulsive Scale (Y-BOCS), (2) a score greater than or equal to 16 on the Yale–Brown Obsessive Compulsive Scale-Self-Report (YBOCS-SR), and (3) being 18 years or older.	“Given the paucity of empirical research examining potential barriers to treatment for OCD, this study was designed to explore this topic in an internet sample of individuals with OC symptoms."	Factors associated with increased delays: Misunderstanding OCD: Lack of information on OCD Perspectives and behaviors surrounding care: Stigma Mistrust in health professionals Denial Fear Doubts on effectiveness of treatments Logistical barriers: Cost of treatment Travel associated with treatment and care Lack of access to resources	Years to seek treatment after onset of symptoms: Mean = 9.72 (SD = 8.30) Logistic and financial barriers to treatment: Worried about cost (57.1 %); Unsure about who to see or where to go (50.4 %); Health insurance not covering treatment (37.8 %); Too inconvenient or time-consuming (31.0 %); Problems with transportation or scheduling (25.2 %); Could not choose the desired provider (22.1 %); Unable to get an appointment (16.2 %) Stigma, shame, and discrimination barriers to treatment: Ashamed of needing help (58.2 %); Wanted to handle it independently (54.5 %); Ashamed of personal problems (53.2 %); Worried about others’ opinions (39.3 %); Afraid of family criticism for seeking help (38.6 %); Uncomfortable discussing problems with a health professional (29.2 %); Afraid of involuntary hospitalization (26.1 %) Treatment perception and satisfaction barriers to treatment: Didn’t think treatment would work (48.9 %); Previous treatment was ineffective (41.6 %); Dissatisfied with available services (30.7 %)
Wahl et al. (2010; Germany) (Wahl et al., 2010) *	Outpatients (who were referred for treatment because of a psychiatric disorder, neurological disorder, or both) seen at 10 different psychiatric practices in South Germany were asked to participate in the study.	"The present study aims to investigate the recognition of OCD, including comorbidity rates, in psychiatric outpatients."	Factors associated with increased delays: Misunderstanding OCD: Misdiagnoses	Only 27.5 % of patients with OCD received this diagnosis by their clinician. As such, over 70 % of patients with OCD did not get adequate treatment
Belloch et al. (2009; Spain) (Belloch et al., 2009)	Adult outpatients with a principal diagnosis of OCD without current comorbid diagnoses who visited a mental-health clinic (included in the network of the public National Health System and located in the outskirts of the city of Valencia, Spain) looking for professional help.	“The specific goals of this study were: first, to examine the extent to which pure OCD patients delay help-seeking, and the extent to which they initially recognize the existence of a problem (that is, insight from the onset of noticeable symptoms); second, to explore the reasons reported by OCD patients to help seek for their disorder; third, to identify the differences between short and long delayers in the help-seeking process; and finally, to analyze the associations among the delay rates and questionnaire measures on obsessionality and thought control strategies.”	Factors associated with increased delays: Misunderstanding OCD: Lack of understanding or recognition of OCD symptoms Lack of information on OCD Perspectives and behaviors surrounding care: Stigma Mistrust in health professionals Denial Fear Doubts on effectiveness of treatments Characteristics of OCD: Content of obsessions Factors associated with decreased delays: Interference with daily life Characteristics of OCD: Symptom characteristics (severity & quality) Motivation from family/friends Demographic factors: Education	“Why did you delay in seeking treatment for your problem?”: I was convinced that the problem was temporary (50 %); I felt I could control problem (34.6 %); I believed my behaviors and/or thoughts were not serious (34.6 %); I felt ashamed by the thought contents (34.6 %); I feared being considered a mentally ill person (30.8 %); I thought it was not a problem requiring professional help or treatment (23.1 %); I was afraid (of the thought contents) (19.2 %); The problem did not interfere in my daily activities (7.69 %); I was afraid someone would tell me I was a bad person for having these thoughts (3.8 %); I feared that if I disclosed the thoughts to someone, the thoughts could come true (3.8 %) “Why did you seek treatment for your problem?”: The problem didn’t disappear, I couldn’t control it (77 %); The problem (thoughts and/or behaviors) interfered with what I was doing (77 %); The problem (thoughts and/or behaviors) became more and more disturbing (73.1 %); The problem (thoughts and/or behaviors) became more frequent (65.4 %); I felt sad (65.4 %); I was afraid of what was happening to me (57.7 %); I believed that my thoughts could come true (57.7 %); I thought I had a serious problem (an illness) (46.2 %); Someone advised me to seek treatment (38.5 %); I thought I was a bad person for having these thoughts (30.8 %) Reasons for “Why did you delay in seeking treatment for your problem?”: I felt ashamed by the thought contents (34.6 %) I was afraid (of the thought contents) (19.2 %) The short-delay group (patients who made formal contact in the first year of initial onset) had a lower educational level (*χ*2(1, 24) = 2.01; P = 0.04), but no other difference was found.
Fullana et al. (2009; New Zealand) (Fullana et al., 2009)	Participants born during a one-year period in Dunedin, New Zealand as part of the Dunedin Multidisciplinary Health and Development Study	“In the present report, we employ a prospective longitudinal design with an unselected birth cohort of individuals. Our research questions were: 1) What is the prevalence of obsessions and compulsions in the community and the associated level of interference? 2) How many people seek help for obsessions/compulsions and what predicts help-seeking behavior? 3) What is the prevalence and temporal stability of specific obsessive-compulsive symptom dimensions? 4) Are these symptom dimensions associated with particular patterns of comorbidity? 5) Is there an association between obsessive-compulsive symptoms in childhood and OCD in adulthood?”	Factors associated with decreased delays: Characteristics of OCD: Content of obsessions Comorbidities Symptom characteristics (severity & quality)	At both ages, help-seeking was associated with having obsessions (age 26, OR = 9.46, 95 % CI = 3.92–22.88; age 32, OR = 7.75, 95 % CI = 2.51–23.95) but not with having compulsions (age 26, odds ratio = 1.89, 95 % CI = .68–5.24; age 32, odds ratio = .26, 95 % CI = .10–.69). Three types of obsessions were associated with seeking help at both ages: hurting/killing someone beloved without wanting to (age 26, odds ratio = 20.12, 95 % CI = 5.82–69.61; age 32, odds ratio = 4.45, 95 % CI = 1.44–13.72), accidently harming someone (age 26, odds ratio = 11.16, 95 % CI = 2.94–42.27; age 32, odds ratio = 3.73, 95 % CI = 1.23–11.33), and shameful thoughts (age 26, odds ratio = 14.77, 95 % CI = 5.92–36.83; age 32, odds ratio = 5.1, 95 % CI = 1.99–13.05). In all, 67 % of study members with OCD who sought help for obsessions or compulsions in the previous year had comorbid major depression.
Cullen et al. (2008; United States) (Cullen et al., 2008)	OCD-affected sibling pairs (and extended, when possible, through affected first- and second-degree relatives) from the OCD Collaborative Genetics Study (OCGS).	“This study sought to explore the factors determining treatment status among family members with OCD using data gathered through the OCD Collaborative Genetics Study. We compared the demographic and clinical factors between subjects with OCD who had received treatment and those who had not.”	Factors associated with increased delays: Demographic factors: Age Marital status Factors associated with decreased delays: Characteristics of OCD: Comorbidities Symptom characteristics (severity & quality) Factors not significantly associated with delays: Demographic factors: Gender Education Income	Subjects who had received treatment were less likely to be married (treated: 55.9 %; untreated: 78 %; *χ*2 = 19.52, df = 2, p < 0.001), and they were more likely to be probands (treated: 44.7 %; untreated: 9.7 %, *χ*2 = 80.111, df = 4, p < 0.001). Gender, educational level and household income were not found to have a significant relationship with treatment status. Subjects who had not received treatment were older (mean current age: treated 33.5 years SD 15.7; untreated 44.3 years SD 18.8; Student’s t-test, p < 0.001). Those who sought treatment for their OCD were more likely to be impaired in several areas: social (treated: 76.5 %; untreated: 49.6 %, *χ*2 = 34.459, df = 1, p < 0.001), academic (treated: 53.7 %; untreated: 22.8 %, *χ*2 = 36.793, df = 1, p < 0.001), occupational (treated: 53.6 %; untreated: 33.9 %, *χ*2 = 14.707, df = 1, p = 0.001) and home (treated: 25.2 %; untreated: 14.4 %, *χ*2 = 17.497, df = 1, p < 0.001). When all factors were considered together in a multivariable model, the number of obsessions (OR = 1.302, CI = 1.077–1.574, p < 0.006), severity of Y-BOCS score (at worst phase) (OR = 1.078, CI = 1.036–1.123, p < 0.001) and presence of co-morbid mania or hypomania (OR = 9.345, CI = 1.052–83.043, p = 0.045) were positively associated with receiving treatment. The mean time to treatment by current age increased as current age increased, ranging from 1.7 years in subjects aged between 7 and 10 years–25.8 years in subjects aged over 50 years (Student’s T-test, p < 0.001).
Grant et al. (2007; United States) (Grant et al., 2007)	Individuals with OCD were recruited from psychiatric treatment settings, including consecutive admissions to an outpatient OCD specialty clinic, inpatient units of a private psychiatric hospital, community mental health centers, general outpatient psychiatric clinics, and private practices in cognitive-behavioral therapy for OCD.	" … we examined the clinical characteristics of a cohort of subjects with an age of OCD onset in middle adulthood or later (onset after age 30) in the hopes of further defining the clinical characteristics of this complex disorder."	Factors associated with increased delays: Characteristics of OCD: Early age of onset Factors not significantly associated with delays: Demographic factors: Gender Family history Characteristics of OCD: Content of obsessions	Patients with OCD with late-onset symptoms were significantly more likely to seek treatment earlier: 5.2 years for the late-onset group, 7.8 years for the young-adult-onset group, and 14.7 years for the early-onset group (F = 18.170; df = 2; p < 0.001). Higher percentage of females in the late-onset OCD group, but no statistical differences in gender between groups (66.7 % female in late-onset group compared to 44.6 % in the young adult group and 56.8 % in the early-onset group; chi-square = 5.645; df = 2; P = 0.059) No difference in family history of OCD in early-onset and late-onset groups. Tendency for late-onset OCD patients to have less contamination, religious, and somatic obsessions, no significant differences in primary obsessions between those with late-onset OCD. Those with late-onset OCD, however, were far less likely to engage in repetitive or checking compulsions.
Torres et al. (2007; United Kingdom) (Torres et al., 2007)	Individuals living in private households in England, Wales, and Scotland who participated in the British Survey of Psychiatric Morbidity of 2000.	“Our aim was to analyze the use of services by people with OCD from a nationally representative sample and compare it with use by those with other neuroses. We also wanted to determine possible differences in services used between genders, individuals with different types of the disorder (only obsessions, only compulsions, or both), and those with or without a comorbid anxiety or depressive disorder. We were also interested in evaluating the appropriateness of therapeutic approaches for individuals who were receiving treatment."	Factors associated with decreased delays: Characteristics of OCD: Comorbidities Factors not significantly associated with delays: Demographic factors: Age Gender Race and ethnicity Characteristics of OCD: Symptom characteristics (severity & quality)	Individuals with OCD and a comorbid disorder were significantly more likely to receive treatment than those with OCD alone (56 % compared with 14 %, p < 0.001). There was a trend for older people with OCD to be more likely to receive treatment, but other sociodemographic factors were not associated with treatment receipt. No significant gender differences were found. In a multivariate analysis, the severity of psychological morbidity (CIS-R total score greater than 18) and lifetime history of suicidal thoughts were no longer associated with receipt of treatment after adjustment for other clinical factors, including presence of comorbid neuroses.
Karadaĝ et al. (2006; Turkey) (Karadaĝ et al., 2006)	Patients at the outpatient psychiatry clinic in the Faculty of Medicine of Pamukkale University registered with a diagnosis of OCD from February 1998 to December 2003. All patients were Muslim.	“This study aimed to investigate the demographic and clinical features of obsessive–compulsive disorder (OCD) and the possible association between obsessive–compulsive symptoms and culture-related characteristics in a sample of Turkish patients with OCD.”	Factors associated with increased delays: Characteristics of OCD: Content of obsessions Comorbidities	Those with religious obsessions had significantly longer delays in starting OCD-specific treatment than those with dirt/contamination obsessions (7.67 (SD = 8.35) vs 4.78 (SD = 7.69) years, U = 1027.0, p = 0.004) Those with sexual obsessions had significantly longer delays in starting OCD-specific treatment than those with dirt/contamination obsessions (7.43 (SD = 7.33) vs 4.78 (SD = 7.69) years, U = 914.0, p = 0.023) Those with religious obsessions had significantly longer delays in seeking first psychiatric help than those with dirt/contamination obsessions (6.89 (SD = 8.05) vs 4.84(SD = 7.68) years, U = 1199.5, p = 0.045) Those with religious obsessions had significantly longer duration of illness than those with dirt/contamination obsessions (10.53 (SD = 8.79) vs 7.66 (SD = 8.52) years, U = 1113.0, p = 0.015) Those with depressive OCD had significantly longer duration of illness than those with non-depressive OCD (9.06 (SD = 8.99) vs 5.80 (SD = 6.46) years, U = 1960.0, p = 0.031) Those with depressive OCD were significantly older at admission than those with non-depressive OCD (35.27 (SD = 12.49) vs 28.45 (SD = 12.81) years, U = 1392.0, p = 0.001) Those with depressive OCD had significantly later age of onset than those with non-depressive OCD (27.16 (SD = 10.75) vs 21.69 (SD = 10.28) years, U = 1426.0, p = 0.002)
Besiroglu et al. (2004; Turkey) (Besiroglu et al., 2004)	Adult subjects from the Psychiatric Outpatient Clinic of the Selcuk University Hospital in Konya who sought health care for OCD for the first time (HCS group) or adult subjects with OCD from the Konya Epidemiologic Study who had not ever sought health care for OCD before (NHCS group).	“The present study aimed to investigate distinctive and overlapping features of non-health care-seekers and health care-seekers with OCD by comparing their illness severity, insight degree, types of obsessions and compulsions, frequency of comorbid diagnoses, and QOL levels. We attempt to describe predictor variables that are associated with the health care seeking behavior for OCD.”	Factors associated with decreased delays: Characteristics of OCD: Content of obsessions Comorbidities Symptom characteristics (severity & quality) Factors not significantly associated with delays: Demographic factors: Age Gender Marital status Education Employment	No significant differences between the groups for age, gender, marital status, education level, or employment status. The mean duration of illness of the non-health care seekers group (NHCS) (12.3 (SD = 5.8)) was significantly longer than the health care seekers group (HCS) (6.0 (SD = 4.3)) (t(df 46): 4.32; p < 0.001). NHCS (21.0 (SD = 4.8)) scored significantly lower than HCS (24.7 (SD = 6.5)) on total Y-BOCS score (t(df 46): 2.19, p < 0.05). NHCS (9.9 (SD = 3.4)) scored significantly lower than HCS (11.8 (SD = 4.3)) on the Y-BOCS obsessive subscale (t(df 46): 3.09, p < 0.005). Compared to HCS, both aggressive and religious obsessions were significantly less identified in NHCS [Aggressive: *χ*2 = 10.04, df = 1, p < 0.005; Religious: *χ*2 = 3.95, df = 1, p < 0.05]. The subjects with comorbid psychiatric diagnosis were significantly less frequent in NHCS (n = 3, 13 %) than in HCS (n = 11, 44 %) (*χ*2 = 5.56, df = 1, p < 0.05). NHCS scored significantly lower than HCS on the Hamilton Depression Rating Scale (HDRS) (NHCS HDRS scores: 8.2 (SD = 6.2); HCS HDRS scores: 12.8 (SD = 3.1); t(df 46): 2.39, p < 0.05).
Caraveo-Anduaga & Colmenares Bermúdez (2004; Mexico) (Caraveo-Anduaga and Bermúdez, 2004)	Adults aged 18–65 years old living permanently or temporarily in private dwellings from the 16 political division areas of Mexico City.	“During 1995, a comprehensive psychiatric epidemiological study was carried out in Mexico City, including the following objectives: 1. to estimate the lifetime and 12-month prevalence of OCD in the adult population aged 18–65 years in Mexico City; 2. to identify the patterns of lifetime comorbidity in subjects with OCD; 3. to study the help-seeking process or the absence of it."	Factors associated with increased delays: Demographic factors: Gender (female) Characteristics of OCD: Comorbidities Factors associated with decreased delays: Characteristics of OCD: Comorbidities	Only 8 % of all OCD cases sought help, and those without any other psychiatric comorbid disorder did not seek help at all. Help was sought mainly from general practitioners and mental health specialists. Women sought help when agoraphobia, depressive episodes, generalized anxiety, social phobia, and dysthymia were comorbid with OCD. Women also sought help from priests and natural healers, when agoraphobia and generalized anxiety were present. Men only sought help from mental health specialists.
Koran et al. (2000; United States) (Koran et al., 2000)	Members of a Kaiser Permanente plan with OCD, served by one of 19 KP outpatient facilities in Northern California, aged six years or older, with continuous membership during the index year (May 1, 1995, through April 30, 1996)	“We report here data on the adequacy of pharmacotherapy for patients with OCD in a large, prepaid HMO.”	Factors associated with increased delays: Demographic factors: Age Factors associated with decreased delays: Demographic factors: Age	Odds of receiving adequate medication were significantly higher in patients between age 18 and 39 years (OR = 3.21, Wald’s *χ*2 = 4.64, p = 0.03) (versus patients 60 or older) and patients between 40 and 59 years of age (OR = 3.76, Wald’s *χ*2 = 5.75, p = 0.02) (versus patients 60 or older). A higher percentage of newly diagnosed children and adolescents (49.4 %) than newly diagnosed adults (27.5 %) had no trial of medication for OCD in the year after diagnosis. A higher percentage of newly diagnosed adults (29.7 %) than of children and adolescents (22.3 %) had an inadequate trial of medication in the 12 months after diagnosis
Stengler-Wenzke et al. (2004; Germany) (Stengler-Wenzke et al., 2004) #	Relatives of OCD patients who were recruited from the specialized outpatient and day clinic for patients with OCD and anxiety disorders at the Department of Psychiatry of the University of Leipzig.	“The present study aims to describe how relatives of people with OCD experience stigmatization and discrimination in their everyday lives.”	Factors associated with increased delays: Misunderstanding OCD: Lack of understanding or recognition of OCD symptoms Lack of information on OCD Misdiagnoses Perspectives and behaviors surrounding care: Stigma Mistrust in health professionals Fear	Four themes from relatives of patient with OCD of stigmatizing experiences: symptom onset, disorder concealment, stigmatization in medical care, and retrospective stigmatization. Delays in accurate OCD diagnosis due to misunderstandings and misdiagnoses by general practitioners and psychologists. OCD behaviors are perceived as bizarre, fueling stigma and causing both patients and their families to struggle with society’s misconceptions about mental illness. Families hide OCD to avoid judgment, with behaviors mislabeled as eccentric quirks, leading to emotional strain and isolation. Families feel neglected and misunderstood by healthcare professionals who may lack empathy or knowledge about OCD. Excluding relatives from treatment due to privacy concerns can lead to feelings of rejection and guilt. Relatives recognize, in hindsight, their own stigmatizing reactions toward the patient, often driven by a lack of understanding, helplessness, and frustration with the disorder’s impact on daily life.

## Data Availability

Data will be made available on request.

## Glossary

AD: Age Diagnosed
ASO: Age of Symptom Onset
ASO-AD: Age of Symptom Onset to Age of Diagnosis
BACE: Barriers to Access Care Evaluation
BTQ: Barriers to Treatment Questionnaire
CIS-R: Clinical Interview Schedule-Revised
CSM: Common-Sense Model of Self-Regulation
DUI: Duration of Untreated Illness
ERP: Exposure and Response Prevention
FOCI-B: Florida Obsessive-Compulsive Inventory
GAD: Generalized Anxiety Disorder
GAF: Global Assessment of Functioning
HBM: Health Belief Model
HCS: Healthcare Seekers
HDRS: Hamilton Depression Rating Scale
IASMHS: Inventory of Attitudes Toward Seeking Mental Health Services
IP: Illness Perceptions
IPQ-O: Illness Perception Questionnaire for OCD
IPQ-R: Illness Perception Questionnaire-Revised
LTT: Latency to Treatment
MDD: Major Depressive Disorder
MR: Median Ratio
NHCS: Non-Healthcare Seekers
NHS: National Health Service
OCD: Obsessive-Compulsive Disorder
OCD-IRS: Obsessive-Compulsive Disorder Intrusiveness Rating Scale
OCI-R: Obsessive-Compulsive Inventory-Revised
PD: Panic Disorder
PRISMA-ScR: Preferred Reporting Items for Systematic Reviews and Meta-Analyses extension for Scoping Reviews
Q-LES-Q-SF: Quality of Life Enjoyment and Satisfaction Questionnaire
SD: Standard Deviation
SSRI: Selective Serotonin Reuptake Inhibitor
USS: Universal Stigma Scale
Y-BOCS: Yale-Brown Obsessive Compulsive Scale
